# *Cannabis sativa* L. Extract Increases COX-1, COX-2 and TNF-α in the Hippocampus of Rats with Neuropathic Pain

**DOI:** 10.3390/molecules30010194

**Published:** 2025-01-06

**Authors:** Joanna Bartkowiak-Wieczorek, Małgorzata Jamka, Radosław Kujawski, Marcin Hołysz, Agnieszka Bienert, Kamila Czora-Poczwardowska, Michał Szulc, Przemysław Mikołajczak, Anna Bogacz, Anna-Maria Wizner, Karolina Wielgus, Ryszard Słomski, Edyta Mądry

**Affiliations:** 1 Physiology Department, Poznan University of Medical Sciences, 6, Święcickiego Street, 60-781 Poznan, Poland; aniabogacz23@o2.pl (A.B.); emadry@ump.edu.pl (E.M.); 2 Department of Paediatric Gastroenterology and Metabolic Diseases, Poznan University of Medical Sciences, Szpitalna Street 27/33, 60-572 Poznan, Poland; mjamka@ump.edu.pl (M.J.); kwielgus@ump.edu.pl (K.W.); 3 Department of Pharmacology, Poznan University of Medical Sciences, 3, Rokietnicka Street, 60-806 Poznan, Poland; radkuj@ump.edu.pl (R.K.); agbienert@ump.edu.pl (A.B.); kczora@ump.edu.pl (K.C.-P.); mszulc@ump.edu.pl (M.S.); przemmik@ump.edu.pl (P.M.); 4 Department of Biochemistry and Molecular Biology, Poznan University of Medical Sciences, 6, Swiecickiego Steet, 60-781 Poznan, Poland; mholysz@ump.edu.pl; 5 Department of Clinical Pharmacy and Biopharmacy, Poznan University of Medical Sciences, 3, Rokietnicka Street, 60-806 Poznan, Poland; anna.usciniak@gmail.com; 6 Department of Biotechnology, Institute of Natural Fibres and Medicinal Plants—National Research Institute, Wojska Polskiego 71B, 60-630 Poznan, Poland; slomski@up.poznan.pl

**Keywords:** CBD, THC, vincristine, lymphocytes, cerebral cortex, NFκB, inflammation

## Abstract

Inflammation is the critical component of neuropathic pain; therefore, this study aimed to assess the potential anti-inflammatory effects of *Cannabis sativa* L. extracts in a vincristine-induced model of neuropathic pain. The effects of different doses (5.0–40.0 mg/kg) of two *Cannabis sativa* L. extracts (B and D) on COX-1, COX-2, TNF-α, and NF-κB mRNA and protein levels were examined in the rat hippocampus, cerebral cortex, and blood lymphocytes. There were statistically significant differences in COX-1, COX-2, and TNF-α mRNA and protein expression in the hippocampus and cerebral cortex, with significant differences in COX-2 and TNF-α in the lymphocytes. Extract D dose-dependently increased COX-1 mRNA and protein in the hippocampus and cortex. In contrast, Extract B dose-dependently increased COX-1 mRNA and decreased COX-2 mRNA (in a dose of 7.5 mg/kg) and TNF-α protein levels in the cortex. *Cannabis sativa* L. extracts significantly influenced the expression of inflammatory genes and proteins, with effects varying based on dose and tissue type. The increased expression of COX-1, COX-2, and TNF-α (in comparison to groups receiving NaCl, vincristine, and gabapentin) in the rat hippocampus and COX-1 in the cerebral cortex suggests that *Cannabis* may have a pro-inflammatory effect. Due to species specificity, the results of our research based on rats require confirmation in humans. However, *Cannabis sativa* should be recommended with caution for treating pain with an inflammatory component.

## 1. Introduction

Neuropathic pain results from harm or abnormal functioning in the part of the nervous system responsible for sensing touch and physical sensations [1,2], and its development is based on an imbalance between pro-inflammatory and anti-inflammatory cytokines [3]. Nerve injury releases inflammatory mediators to modulate the excitability of nerve cells, leading to peripheral or central sensitization of nervous system structures [4].

Vincristine chemotherapy contributes to the development of peripheral neuropathy, manifested by increased sensitivity to painful (hyperalgesia) and non-painful (allodynia) stimuli [5]. Persistent inflammation in both the peripheral and central nervous systems significantly contributes to the maintenance and development of neuropathic pain [6]. Vincristine damages the nervous system by inhibiting tubulin polymerization, leading to changes in the microtubule structure and cessation of cell division in neurons causing glial cell disorganization [6]. Increased astrocytes and microglial cell activity in the spinal cord occur after peripheral nervous system damage, manifested by morphological changes and increased secretion of pro-inflammatory mediators, with tumour necrosis factor-alpha (TNF-α) playing a key role [7].

TNF-α induces NF-κB activation, increasing the secretion of other inflammatory cytokines, as well as cyclooxygenase-2 (COX-2) expression, which plays a significant role in vincristine-induced neuropathy by catalyzing arachidonic acid metabolism and producing pro-inflammatory prostaglandins [8]. Inflammation is involved in the early stages of the body’s defence reaction, but its exacerbation contributes to cell apoptosis and, consequently, increased pain perception, including neuropathic pain [9].

Cyclooxygenases (COX-1, COX-2) and TNF-α are essential in mediating inflammation and developing neuropathic pain [10,11]. Oxidation of the endogenous cannabinoid, 2-arachidonoylglycerol (2-AG), by COX-2 leads to the production of glyceryl ester of prostaglandin H(2) (PGH(2)-G) in the presence of calcium and prostaglandin E2 ethanolamide [12,13]. Increased COX-1 activity in rat spinal cord glial cells correlates with local mechanical hypersensitivity, but COX-1 expression in the spinal cord in peripheral inflammation remains unchanged [14].

*Cannabis sativa* (CSL) extracts exhibit pleiotropic effects and mutual interactions between various phytocannabinoids and between phytocannabinoids and terpenes, generating unique effects compared to the action of a single chemical component, a phenomenon known as the “entourage effect” [15]. Phytocannabinoids such as CBD (cannabidiol) and THC (Δ9-tetrahydrocannabinol) interact with cannabinoid receptors CB1 and CB2 to regulate inflammatory and analgesic processes including neuropathic pain [16,17]. In our study, we utilized two different varieties of *Cannabis sativa* extracts. The choice of the two varieties of CSL—Extract B (variety Tygra) and Extract D (variety Dora)—in the study is grounded in their distinct phytochemical profiles, which suggest different mechanisms and potential therapeutic effects. Extract B, characterized by a higher cannabidiol (CBD) content, appears particularly effective in managing neuropathic pain. Extract D is distinguished by a unique blend of cannabinoids and terpenes, including cannabigerol (CBG). This extract might exert a more broad-spectrum effect, possibly due to the synergistic interactions between these compounds. The presence of CBG and other terpenes in Extract D may contribute to an amplified pain-relieving effect, which is particularly relevant in cases where complex pain management is required.

Regarding cannabinoid content, HPLC characterization revealed that while both extracts share a similar overall cannabinoid profile, Extract D displays a slightly higher ∆9-THC content, suggesting a potential difference in psychoactive versus therapeutic effects between the two extracts. This variation might influence the clinical application of each extract, where Extract B could be preferred for conditions requiring minimal psychoactive effects and stronger anti-inflammatory action, and Extract D for cases where a broader therapeutic range is beneficial [17,18]. The choice of both extracts allows for a comparative analysis that can elucidate their relative efficacy in treating neuropathic pain. The study provides insights into how these extracts modulate pain pathways differently by examining dose–response relationships and therapeutic endpoints across different experimental conditions. Integrating such data is crucial for optimizing treatment strategies and understanding the nuanced roles of cannabinoids and terpenes in pain management.

This study aimed to assess the potential anti-inflammatory effects of CSL extracts administered to rats with neuropathic pain evoked by vincristine by analyzing COX-1, COX-2, TNF-α, and NF-κB mRNA and protein expression in the hippocampus, prefrontal cortex, and lymphocytes.

## 2. Results

### 2.1. Results and Composition of Cannabinoid Extraction in Extracts B and D [17,18]

This study evaluated different cannabinoid extraction methods using water- and ethanol-based solvents to measure cannabidiol (CBD) and Δ9-tetrahydrocannabinol (Δ9-THC) content in industrial hemp. Extract B (Tygra variety) and Extract D (Dora variety) were analyzed using High-Performance Liquid Chromatography (HPLC).

Extract B (Tygra): Prepared via maceration using 80% ethanol, Extract B achieved a CBD content of 0.064% in water-based extractions, increasing to 0.036% Δ9-THC with 80% ethanol. Further compositional analysis revealed the following:

- CBD-A: 1.2 mg/g;
- CBD: 220.2 mg/g;
- Δ9-THC-A: 0.1 mg/g;
- Δ9-THC: 15.5 mg/g.

This Polish monoecious industrial hemp variety, cultivated at the Institute of Natural Fibers and Medicinal Plants, demonstrated high CBD yields and low Δ9-THC levels, consistent with industrial hemp standards.

Extract D (KC Dora): Using ultrasound-assisted extraction with 96% ethanol, Extract D exhibited higher cannabinoid yields, with Δ9-THC content reaching 0.057%, the highest among the tested extraction methods. Its detailed composition is as follows:

- CBD-A: 1.2 mg/g;
- CBD: 215.2 mg/g;
- Δ9-THC-A: 0.15 mg/g;
- Δ9-THC: 13.3 mg/g.

This Hungarian monoecious fibrous hemp variety, bred at Agromag Kft, also highlighted ethanol extraction with ultrasound as an effective method for maximizing cannabinoid recovery.

### 2.2. Neuropathic Pain Induction and Treatment

Two behavioural tests were conducted to confirm the induction of neuropathic pain in rats: the tail-flick test and the von Frey test. Differences were observed between the vincristine (VK) and NaCl groups across all time points in the tail-flick test, with the vincristine (VK) group showing significantly increased pain sensitivity compared to control animals in the von Frey test.

**Figure 1:**
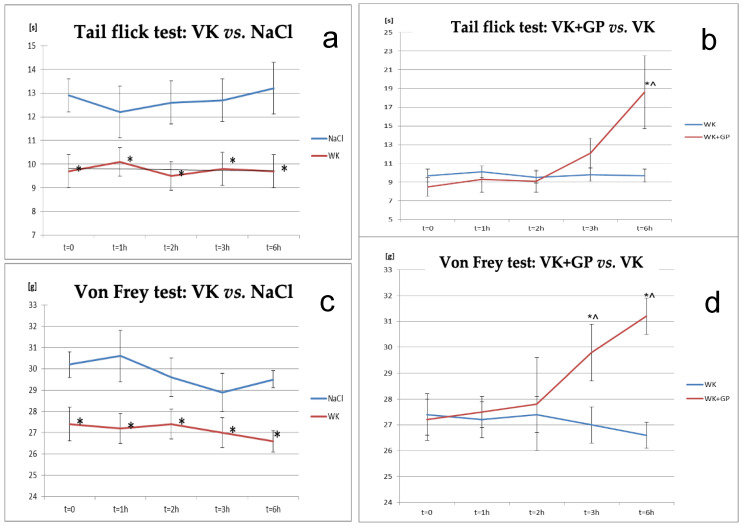
Changes in pain response in the tail-flick test: (**a**) tail-flick test: Vincristine (VK) caused a significant reduction in nociceptive response time compared to the control group (NaCl), reflecting increased thermal hyperalgesia; (**b**) tail-flick test: Adding gabapentin (VK + GP) reversed hyperalgesia caused by vincristine, showing significant analgesic effects over time and von Frey tests in animals after administration of vincristine and vincristine and gabapentin compared to the control group (NaCl) and compared to the group of animals receiving vincristine alone: (**c**) von Frey test: VK significantly reduced mechanical pain thresholds compared to NaCl, indicating mechanical allodynia; (**d**) von Frey test: Gabapentin (VK + GP) increased pain thresholds compared to VK alone, mitigating allodynia. *—statistically significant differences compared to the control group (NaCl; *p* < 0.05); ^—statistically significant differences for the respective group compared to t = 0 (*p* < 0.05).

The antinociceptive effect of gabapentin (GP) was confirmed in both the tail-flick and von Frey tests in rats receiving both vincristine and gabapentin (VK + GP), compared to the vincristine-only group (VK). A significant difference was observed between the VK and VK + GP groups after 6 h of gabapentin administration, with pain sensitivity beginning to increase after 3 h in the tail-flick test. Additionally, at the sixth hour, the effect was statistically significant at the initial measurement point (T0) for the VK + GP group relative to VK alone (*p* < 0.05). In the von Frey test, significant differences were observed between the VK + GP and VK groups at 3 and 6 h post-administration (Figure 1).

**Figure 2:**
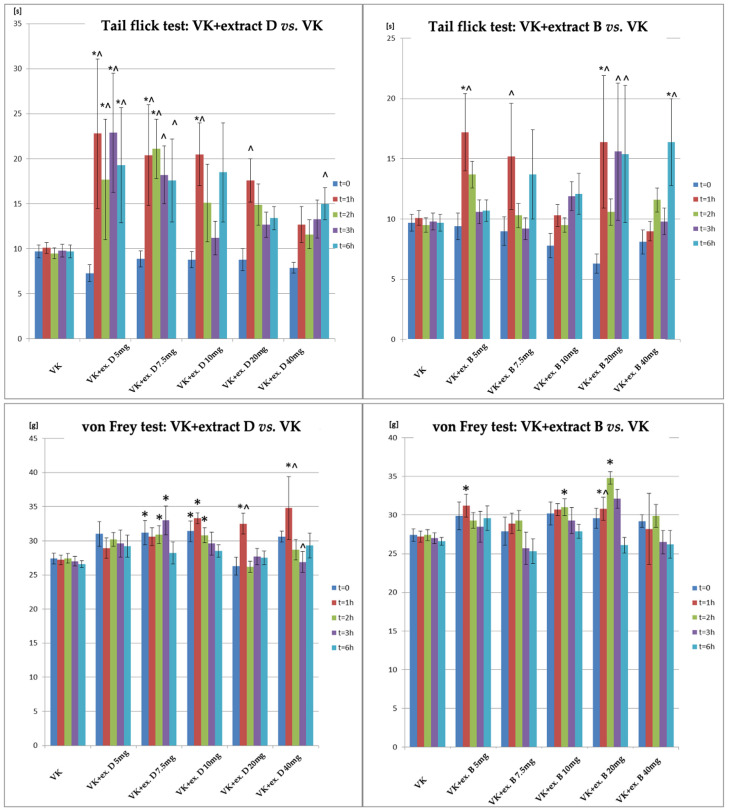
Changes in the pain response in the tail-flick and von Frey tests for animals administered vincristine and either Extract D or Extract B at varying doses (5–40 mg/kg, p.o.) compared to the vincristine-only group. *—statistically significant differences compared to VK (*p* < 0.05); ^—statistically significant differences for the respective group compared to t = 0 (*p* < 0.05).

A comparison of the antinociceptive effects in the tail-flick and von Frey tests is shown in Figure 2. In the tail-flick test, Extract B increased the response time to thermal stimuli, with significant effects seen at 1 h after 5 mg/kg and 20 mg/kg doses. Significant results were observed at 6 h with 40 mg/kg. Extract D showed significant effects at 5 mg/kg (1, 2, 3, 6 h), 7.5 mg/kg (1–2 h), and 10 mg/kg (1 h), with higher doses showing significant effects at 6 h. In the von Frey test, Extract D showed significant effects at 7.5 mg/kg, 10 mg/kg, 20 mg/kg, and 40 mg/kg doses, with the most notable effect at 1 h after 10 mg/kg, 20 mg/kg, and 40 mg/kg. Significant increases were observed 2 h after 7.5 mg/kg and 10 mg/kg, and 3 h after 7.5 mg/kg. For Extract B, significant effects were seen at 20 mg/kg after 1, 2, and 3 h, and at 5 mg/kg after 1 h and 10 mg/kg after 2 h (Figure 2).

### 2.3. COX-1 Protein and mRNA Expression in Rat Brain and Blood Lymphocytes

**Figure 3:**
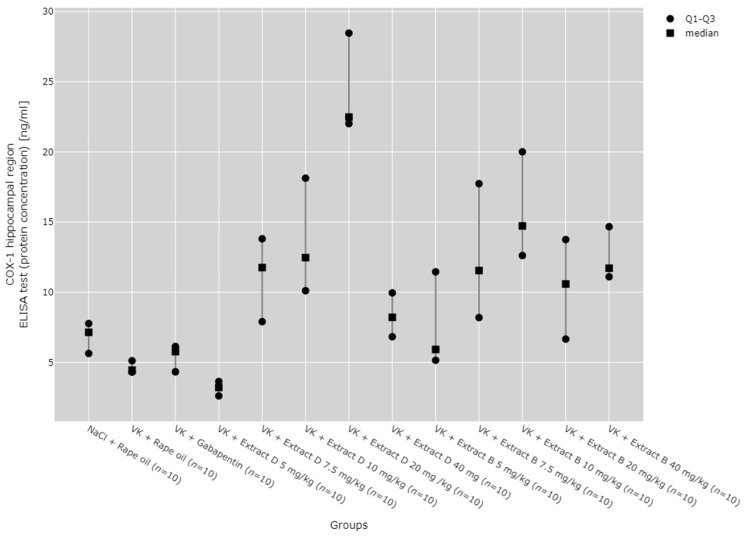
Comparison of COX-1 protein concentrations in the hippocampal region between groups, measured by ELISA assay. Statistical analysis was performed using the Kruskal–Wallis test (*p* < 0.001). Post hoc test results: VK + Extract D 5 mg/kg vs. VK + Extract D 7.5 mg/kg (*p* = 0.026), VK + Extract D 5 mg/kg vs. VK + Extract D 10 mg/kg (*p* = 0.012), VK + Extract D 5 mg/kg vs. VK + Extract D 20 mg/kg (*p* < 0.001), VK + Extract D 5 mg/kg vs. VK + Extract B 7.5 mg/kg (*p* = 0.003), VK + Extract D 5 mg/kg vs. VK + Extract B 10 mg/kg (*p* < 0.001), VK + Extract D 5 mg/kg vs. VK + Extract B 40 mg/kg (*p* = 0.001), VK + Extract B 20 mg/kg vs. NaCl + Rape Oil (*p* = 0.021), VK + Extract D 20 mg/kg vs. VK + Rape Oil (*p* < 0.001), VK + Extract D 20 mg/kg vs. VK + gabapentin (*p* = 0.002), VK + Extract B 10 mg/kg vs. VK + Rape Oil (*p* = 0.004), VK + Extract B 10 mg/kg vs. VK + gabapentin (*p* = 0.019) (only statistically significant results are presented).

**Figure 4:**
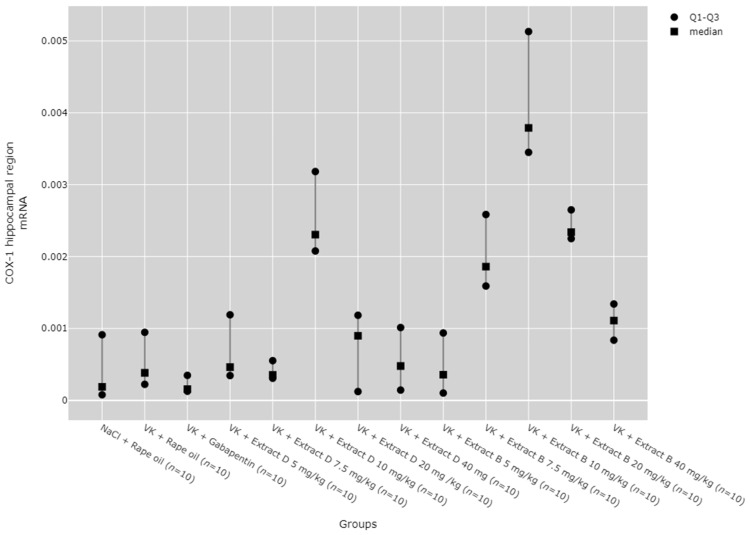
Comparison of COX-1 mRNA expression in the hippocampal region between groups. Statistical analysis was performed using the Kruskal–Wallis test (*p* < 0.001). Post hoc test results: VK + Extract D 10 mg/kg vs. NaCl + Rape Oil (*p* = 0.048), VK + Extract D 10 mg/kg vs. VK + gabapentin (*p* = 0.006), VK + Extract B 7.5 mg/kg vs. VK + gabapentin (*p* = 0.009), VK + Extract B 10 mg/kg vs. NaCl + Rape Oil (*p* = 0.001), VK + Extract B 10 mg/kg vs. VK + Rape Oil (*p* = 0.010), VK + Extract B 10 mg/kg vs. VK + gabapentin (*p* < 0.001), VK + Extract B 10 mg/kg vs. VK + Extract D 5 mg/kg (*p* = 0.035), VK + Extract B 10 mg/kg vs. VK + Extract D 7.5 mg/kg (*p* = 0.004), VK + Extract B 10 mg/kg vs. VK + Extract D 20 mg/kg (*p* = 0.027), VK + Extract B 10 mg/kg vs. VK + Extract D 40 mg/kg (*p* = 0.003), VK + Extract B 10 mg/kg vs. VK + Extract B 5 mg/kg (*p* = 0.002), VK + Extract B 20 mg/kg vs. NaCl + Rape Oil (*p* = 0.034), VK + Extract B 20 mg/kg vs. VK + gabapentin (*p* = 0.004) (only statistically significant results are presented).

A statistically significant variation was found in COX-1 protein and mRNA levels in both the hippocampus and cerebral cortex among all experimental groups (*p* < 0.001, Figure 1, Figure 2, Figure 3 and Figure 4).

Subsequent post hoc analysis indicated that, within the hippocampus, COX-1 protein expression at the 5 mg/kg dose of Extract D (VK + Extract D 5 mg/kg) was significantly lower than at higher doses of the same extract and than at higher doses of Extract B (*p* < 0.05). In contrast, COX-1 protein expression at the 20 mg/kg dose of extract D (VK + Extract D 20 mg/kg) was significantly greater than in the NaCl (*p* = 0.021), vincristine (*p* < 0.001), and vin + gabapentin groups (*p* = 0.002). The 10 mg/kg dose of Extract B (Extract B 10 mg/kg) also showed a significantly higher COX-1 protein level compared to the vincristine group (VK + Rape Oil, *p* = 0.004) and the gabapentin group (VK + gabapentin, *p* = 0.019) (Figure 3).

Regarding COX-1 mRNA expression in the hippocampus, an increase was observed with the 10 mg/kg doses of both Extracts D (VK + Extract D 10 mg/kg) and B (VK + Extract B 10 mg/kg), as well as with the 20 mg/kg dose of Extract B (VK + Extract B 20 mg/kg), when compared to the NaCl (NaCl + Rape Oil) and gabapentin (VK + gabapentin) groups (*p* < 0.05). Additionally, the 10 mg/kg dose of Extract B (Extract B 10 mg/kg) caused a rise in COX-1 expression compared to the vincristine group (VK + Rape Oil, *p* = 0.010), lower doses of Extract D (5 mg/kg (*p* = 0.035) and 7.5 mg/kg (*p* = 0.004), and higher doses of Extract D (20 mg/kg (*p* = 0.027) and 40 mg/kg (*p* = 0.003). Furthermore, a 7.5 mg/kg dose of Extract B led to a marked increase in COX-1 expression compared to the gabapentin group (VK + gabapentin, *p* = 0.009). At the same time, the 10 mg/kg dose of VK + Extract B significantly increased COX-1 mRNA levels in comparison to the 5 mg/kg dose of VK + Extract D (*p* = 0.035) (Figure 4).

**Figure 5:**
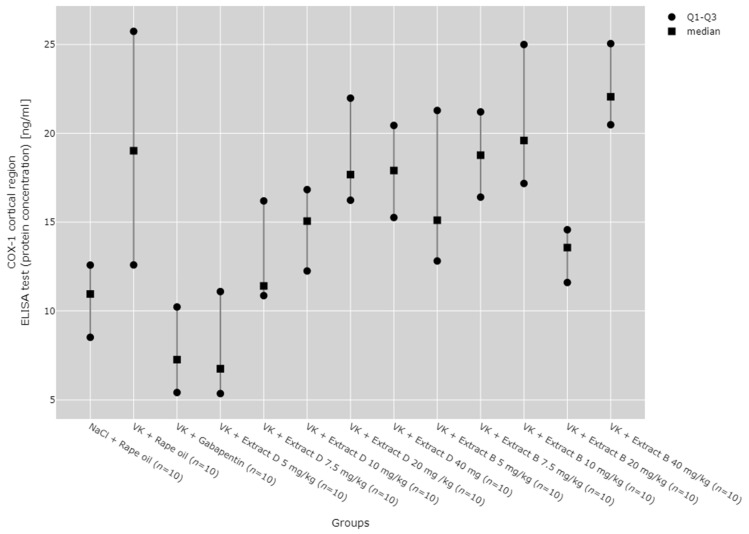
Comparison of COX-1 protein concentrations in the cortical region between groups, measured by ELISA assay. Statistical analysis was performed using the Kruskal–Wallis test (*p* < 0.001). Post hoc test results: VK + Extract B 40 mg/kg vs. NaCl + Rape Oil (*p* = 0.008), VK + Extract B 40 mg/kg vs. VK + gabapentin (*p* = 0.001), VK + Extract B 40 mg/kg vs. VK + Extract D 5 mg/kg (*p* = 0.002) (only statistically significant results are presented).

In the cerebral cortex, the COX-1 protein level significantly increased with the administration of Extract B at a 40 mg/kg dose (VK + Extract B 40 mg/kg), when compared to the NaCl (NaCl + Rape Oil, *p* = 0.008) and gabapentin (VK + gabapentin, *p* = 0.001) groups, as well as to the 5 mg/kg dose of Extract D (*p* = 0.002, Figure 5).

**Figure 6:**
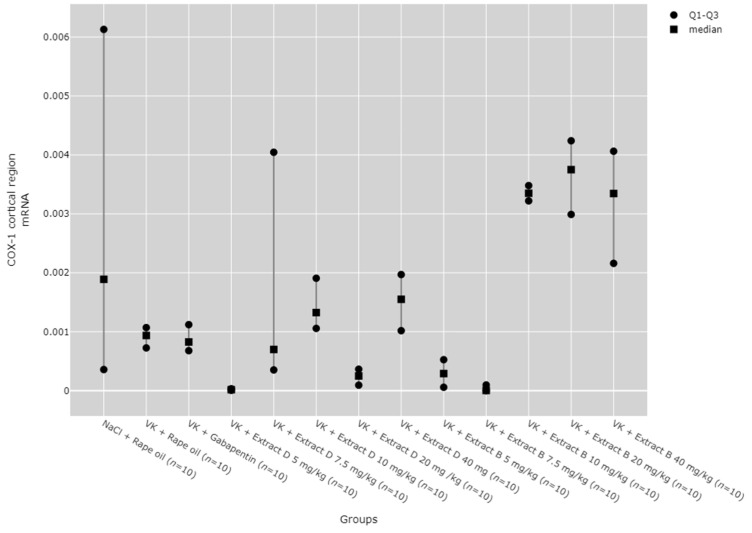
Comparison of COX-1 mRNA expression in the cortical region between groups. Statistical analysis was performed using the Kruskal–Wallis test (*p* < 0.001). Post hoc test results: VK + Extract B 10 mg/kg vs. VK + Extract D 5 mg/kg (*p* = 0.001), VK + Extract B 10 mg/kg vs. VK + Extract B 7.5 mg/kg (*p* = 0.018), VK + Extract B 20 mg/kg vs. VK + Extract D 5 mg/kg (*p* = 0.001), VK + Extract B 20 mg/kg vs. VK + Extract B 7.5 mg/kg (*p* = 0.013), VK + Extract B 40 mg/kg vs. VK + Extract D 5 mg/kg (*p* = 0.011) (only statistically significant results are presented).

COX-1 mRNA expression in the cerebral cortex was notably higher with Extract B at doses of 10 mg/kg (VK + Extract B 10 mg/kg, *p* = 0.001), 20 mg/kg (VK + Extract B 20 mg/kg, *p* = 0.001), and 40 mg/kg (VK + Extract B 40 mg/kg, *p* = 0.011) compared to the 5 mg/kg dose of Extract D (Extract D 5 mg/kg). The 10 mg/kg (VK + Extract B 10 mg/kg, *p* = 0.018) and 20 mg/kg (VK + Extract B 20 mg/kg, *p* = 0.013) doses of Extract B also produced a substantial increase in COX-1 mRNA levels in the cerebral cortex compared to the 7.5 mg/kg dose (Figure 6).

**Figure 7:**
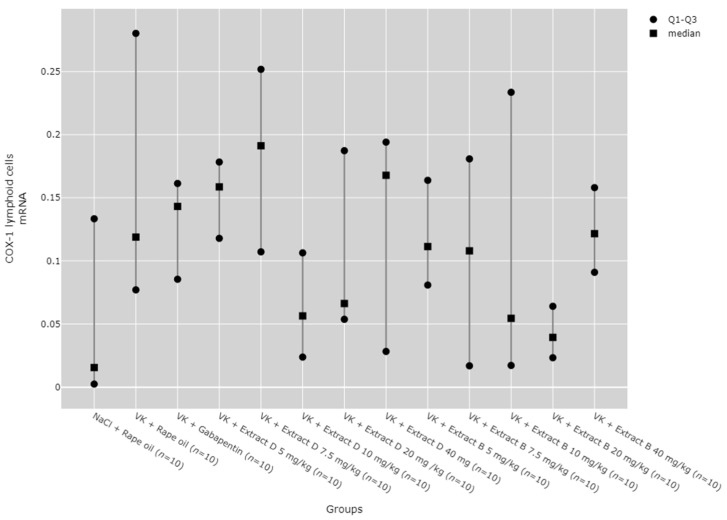
Comparison of COX-1 mRNA expression in the lymphoid cells between groups. Statistical analysis was performed using the Kruskal–Wallis test (*p* = 0.09).

No statistically significant differences between the groups were observed in COX-1 mRNA expression in lymphoid cells (Figure 7).

The dose-dependent effects of the extracts were assessed using the Jonckheere–Terpstra test, revealing that Extract D dose-dependently increased COX-1 protein in the hippocampus from a dose of 5 mg/kg to 20 mg/kg (*p* = 0.0023, Figure 1) and in the cerebral cortex from 5 mg/kg to 40 mg/kg (*p* = 0.0012, Figure 3). Trend analysis for COX-1 mRNA levels in the cerebral cortex showed significant differences between different doses of Extract B (VK + Extract B) (*p* = 0.023, Figure 4) and a dose-dependent increase for Extract D except at the 20 mg/kg dose (VK + Extract D 20 mg/kg, *p* = 0.015, Figure 4).

### 2.4. COX-2 Protein and mRNA Expression in the Rat Brain and Blood Lymphocytes

**Figure 8:**
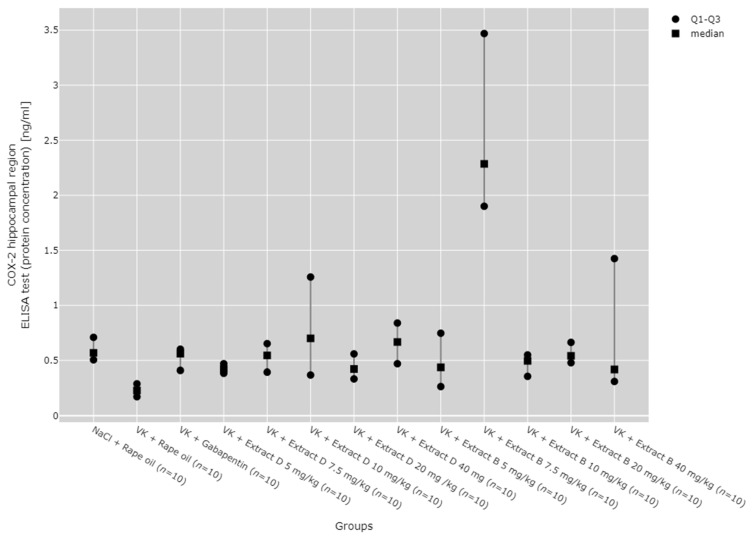
Comparison of COX-2 protein concentrations in the hippocampal region between groups, measured by ELISA assay. Statistical analysis was performed using the Kruskal–Wallis test (*p* < 0.001). Post hoc test results: VK + Extract D 40 mg/kg vs. VK + Rape Oil (*p* = 0.031), VK + Extract B 7.5 mg/kg vs. VK + Rape Oil (*p* < 0.001), VK + Extract B 7.5 mg/kg vs. VK + Extract D 5 mg/kg (*p* < 0.001), VK + Extract B 7.5 mg/kg vs. VK + Extract D 20 mg/kg (*p* = 0.014), VK + Extract B 7.5 mg/kg vs. VK + Extract B 5 mg/kg (*p* = 0.014), VK + Extract B 7.5 mg/kg vs. VK + Extract B 10 mg/kg (*p* = 0.038) (only statistically significant results are presented).

**Figure 9:**
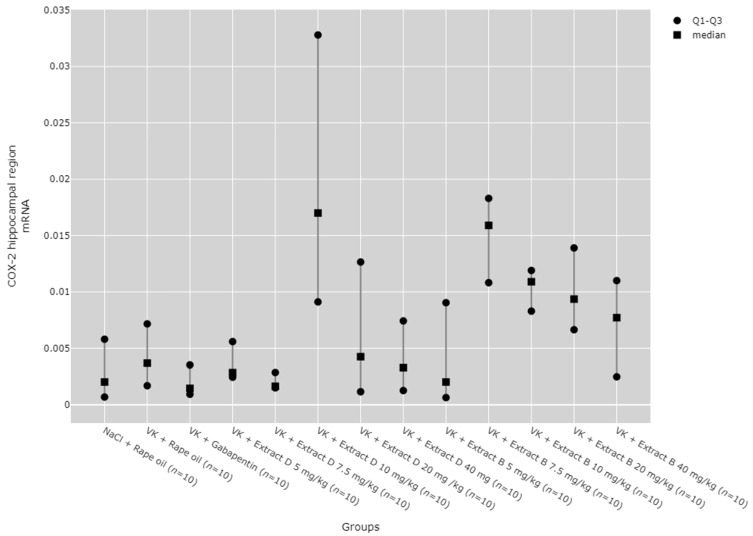
Comparison of COX-2 mRNA expression in the hippocampal region between groups. Statistical analysis was performed using the Kruskal–Wallis test (*p* < 0.001). Post hoc test results: VK + Extract D 10 mg/kg vs. VK + gabapentin (*p* = 0.024), VK + Extract D 10 mg/kg vs. VK + Extract D 7.5 mg/kg (*p* = 0.017), VK + Extract B 7.5 mg/kg vs. VK + gabapentin (*p* = 0.010), VK + Extract B 7.5 mg/kg vs. VK + Extract D 7.5 mg/kg (*p* = 0.007) (only statistically significant results are presented).

**Figure 10:**
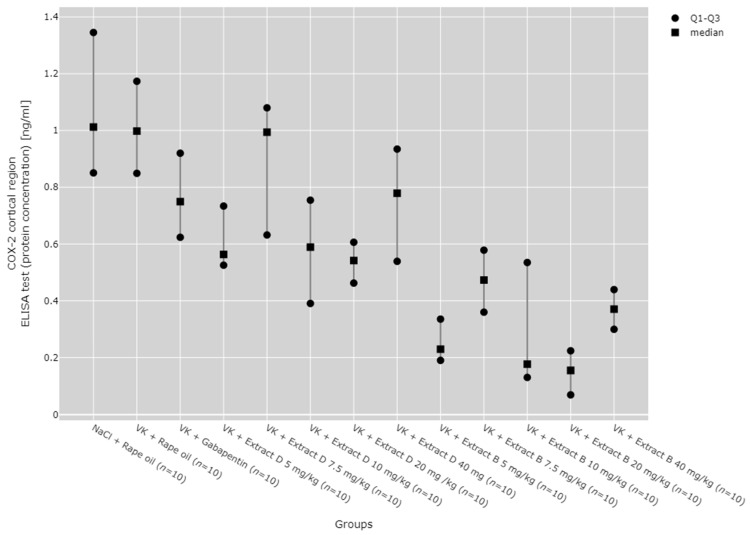
Comparison of COX-2 protein concentrations in the cortical region between groups, measured by ELISA. Statistical analysis was performed using the Kruskal–Wallis test (*p* < 0.001). Post hoc test results: VK + Extract B 5 mg/kg vs. NaCl + Rape Oil (*p* < 0.001), VK + Extract B 5 mg/kg vs. VK + Rape Oil (*p* < 0.001), VK + Extract B 5 mg/kg vs. VK + gabapentin (*p* = 0.014), VK + Extract B 5 mg/kg vs. VK + Extract D 7.5 mg/kg (*p* = 0.014), VK + Extract B 10 mg/kg vs. NaCl + Rape Oil (*p* = 0.007), VK + Extract B 10 mg/kg vs. VK + Rape Oil (*p* = 0.008), VK + Extract B 20 mg/kg vs. NaCl + Rape Oil (*p* < 0.001), VK + Extract B 20 mg/kg vs. VK + Rape Oil (*p* < 0.001), VK + Extract B 20 mg/kg vs. VK + gabapentin (*p* = 0.005), VK + Extract B 20 mg/kg vs. VK + Extract D 7.5 mg/kg (*p* = 0.005), VK + Extract B 40 mg/kg vs. NaCl + Rape Oil (*p* = 0.037), VK + Extract B 40 mg/kg vs. VK + Rape Oil (*p* = 0.049) (only statistically significant results are presented).

There were statistically significant differences between groups in COX-2 protein and mRNA levels in the hippocampus (*p* < 0.001), cerebral cortex (*p* < 0.001), and lymphocytes (*p* = 0.014, Figure 6, Figure 7, Figure 8, Figure 9 and Figure 10).

Post hoc results showed that COX-2 protein expression in the hippocampus was significantly higher after administration of 7.5 mg/kg of Extract B (VK + Extract B 7.5 mg/kg) compared to the vincristine group (VK + Rape Oil, *p* < 0.001), as well as the 5 mg/kg dose of both Extract B (VK + Extract B 5 mg/kg, *p* = 0.0136) and Extract D (VK + Extract D 5 mg/kg, *p* < 0.001). It was also higher compared to the 10 mg/kg dose of Extract B (VK + Extract B 10 mg/kg, *p* = 0.038) and the 20 mg/kg dose of Extract D (VK + Extract D 20 mg/kg, *p* = 0.014). Extract D at 40 mg/kg (VK + Extract D 40 mg/kg) also significantly increased COX-2 protein expression compared to the vincristine group (VK + Rape Oil, *p* = 0.031, Figure 8).

The 10 mg/kg dose of Extract D (VK + Extract D 10 mg/kg) significantly increased COX-2 mRNA expression in the hippocampus compared to the gabapentin group (VK + gabapentin, *p* = 0.024) and the 7.5 mg/kg dose of Extract D (VK + Extract D 7.5 mg/kg, *p* = 0.017). Similarly, the 7.5 mg/kg dose of Extract B (VK + Extract B 7.5 mg/kg) significantly increased COX-2 mRNA expression in the hippocampus compared to gabapentin (VK + gabapentin, *p* = 0.0101) and the same dose of Extract D (VK + Extract D 7.5 mg/kg, *p* = 0.007, Figure 9).

COX-2 protein expression in the cerebral cortex was reduced after 5 mg/kg (VK + Extract B 5 mg/kg), 10 mg/kg (VK + Extract B 10 mg/kg), 20 mg/kg (VK + Extract B 20 mg/kg), and 40 mg/kg (VK + Extract B 40 mg/kg) doses of Extract B compared to the NaCl group (NaCl + Rape Oil, *p* < 0.001; *p* = 0.007; *p* < 0.001; *p* = 0.0370) and the vincristine group (VK + Rape Oil, *p* < 0.001; *p* = 0.008; *p* < 0.001; *p* = 0.049). Additionally, the 5 mg/kg (VK + Extract B 5 mg/kg) and 20 mg/kg (VK + Extract B 20 mg/kg) doses of Extract B reduced COX-2 protein expression in the cortex compared to the gabapentin group (VK + gabapentin, *p* = 0.014; *p* = 0.005) and the 7.5 mg/kg dose of Extract D (Extract D 7.5 mg/kg, *p* = 0.014; *p* = 0.005; Figure 10).

**Figure 11:**
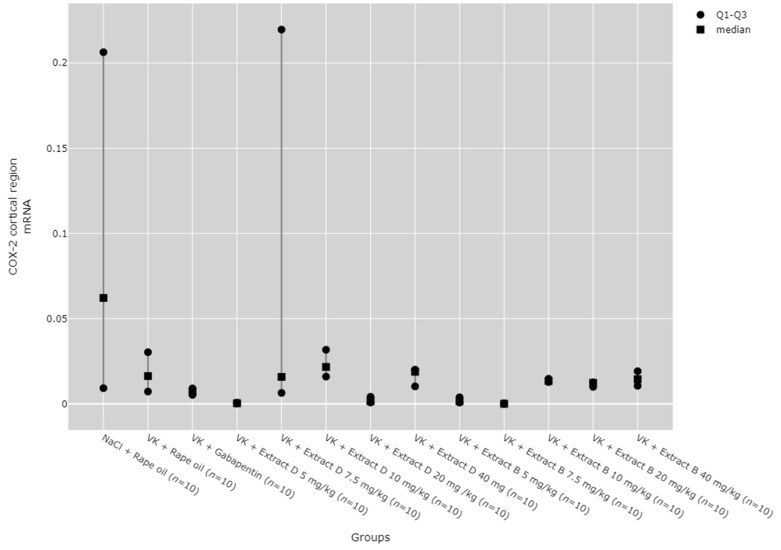
Comparison of COX-2 mRNA expression in the cortical region between groups. Statistical analysis was performed using the Kruskal–Wallis test (*p* < 0.001). Post hoc test results: VK + Extract B 7.5 mg/kg vs. NaCl + Rape Oil (*p* = 0.036), VK + Extract B 7.5 mg/kg vs. VK + Extract D 10 mg/kg (*p* = 0.016) (only statistically significant results are presented).

COX-2 mRNA expression in the cerebral cortex was significantly reduced by the 7.5 mg/kg dose of Extract B (VK + Extract B 7.5 mg/kg) compared to the NaCl group (NaCl + Rape Oil, *p* = 0.036) and the 10 mg/kg dose of Extract D (VK + Extract D 10 mg/kg, *p* = 0.016; Figure 11).

**Figure 12:**
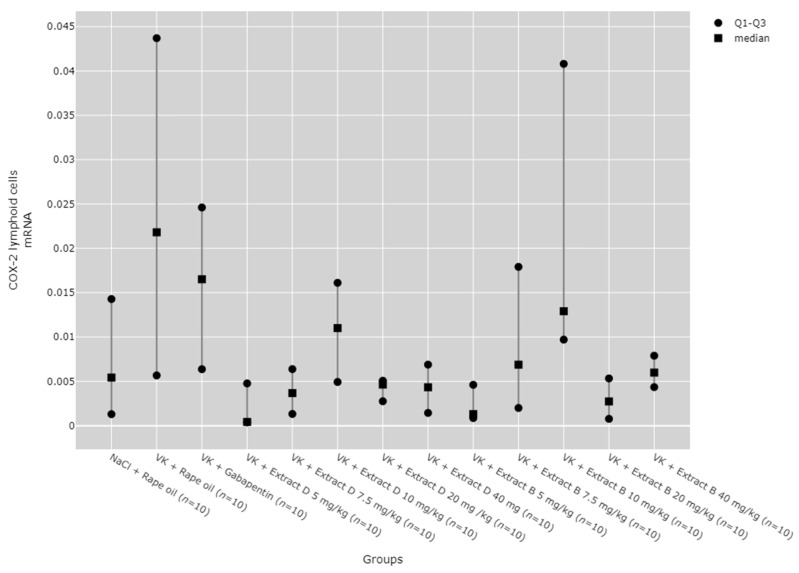
Comparison of COX-2 mRNA expression in the lymphoid cells between groups. Statistical analysis was performed using the Kruskal–Wallis test (*p* = 0.0139).

Statistically significant differences between groups were also detected for COX-2 mRNA expression in lymphoid cells (*p* = 0.139); however, no significant results were found in the post hoc tests (Figure 12).

The trend analyses revealed no significant dose-dependent changes in COX-2 protein or mRNA levels in the hippocampus for Extract D and Extract B (Figure 8 and Figure 9). Likewise, neither extract exerted dose-dependent effects on COX-2 protein expression in the cortex (Figure 10), with the 7.5 mg/kg dose of Extract B significantly reducing COX-2 mRNA in the cortex (VK + Extract B 7.5 mg/kg, *p* = 0.009, Figure 11).

### 2.5. TNF-α Protein and mRNA Expression in the Rat Brain and Blood Lymphocytes

**Figure 13:**
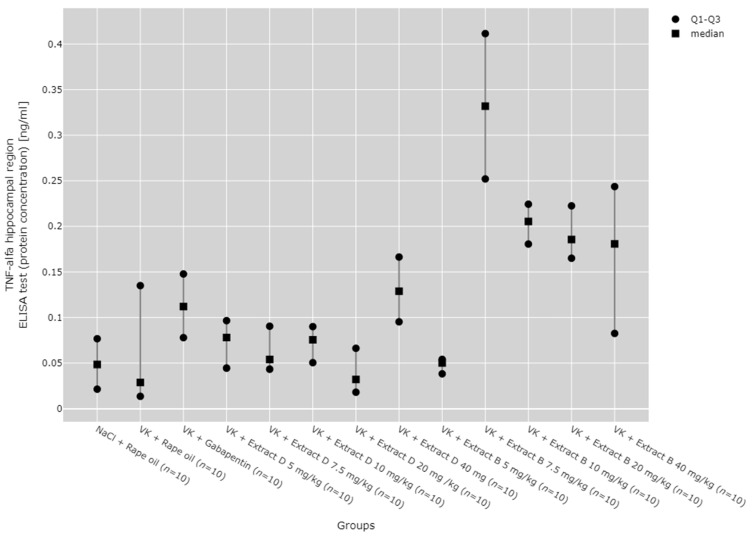
Comparison of TNF-α protein concentrations in the hippocampal region between groups, measured by ELISA assay. Statistical analysis was performed using the Kruskal–Wallis test (*p* < 0.001). Post hoc test results: VK + Extract B 7.5 mg/kg vs. NaCl + Rape Oil (*p* = 0.004), VK + Extract B 7.5 mg/kg vs. VK + Rape Oil (*p* = 0.006), VK + Extract B 7.5 mg/kg vs. VK + Extract D 5 mg/kg (*p* = 0.006), VK + Extract B 7.5 mg/kg vs. VK + Extract D 7.5 mg/kg (*p* = 0.025), VK + Extract B 7.5 mg/kg vs. VK + Extract D 10 mg/kg (*p* = 0.017), VK + Extract B 7.5 mg/kg vs. VK + Extract D 20 mg/kg (*p* = 0.001), VK + Extract B 7.5 mg/kg vs. VK + Extract B 5 mg/kg (*p* = 0.004) (only statistically significant results are presented).

**Figure 14:**
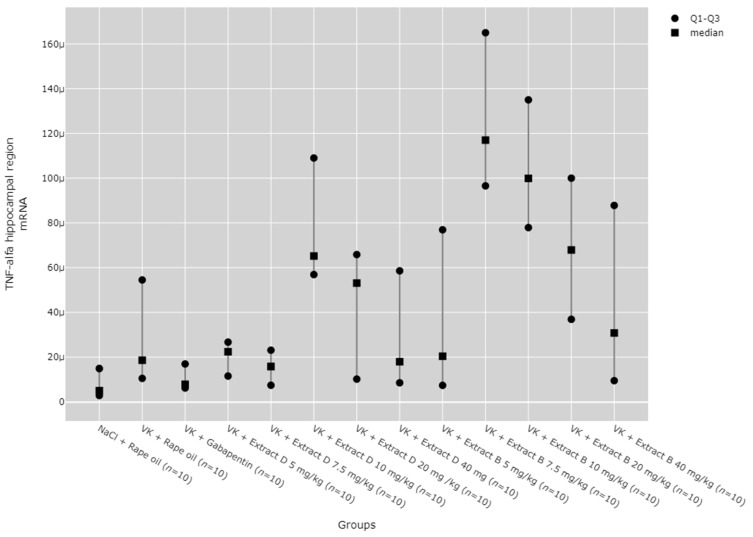
Comparison of TNF-α mRNA expression in the hippocampal region between groups. Statistical analysis was performed using the Kruskal–Wallis test (*p* < 0.001). Post hoc test results: VK + Extract B 7.5 mg/kg vs. NaCl + Rape Oil (*p* = 0.002), VK + Extract B 7.5 mg/kg vs. VK + gabapentin (*p* = 0.001), VK + Extract B 7.5 mg/kg vs. VK + Extract D 7.5 mg/kg (*p* = 0.014), VK + Extract B 10 mg/kg vs. NaCl + Rape Oil (*p* = 0.026), VK + Extract B 10 mg/kg vs. VK + gabapentin (*p* = 0.008) (only statistically significant results are presented).

**Figure 15:**
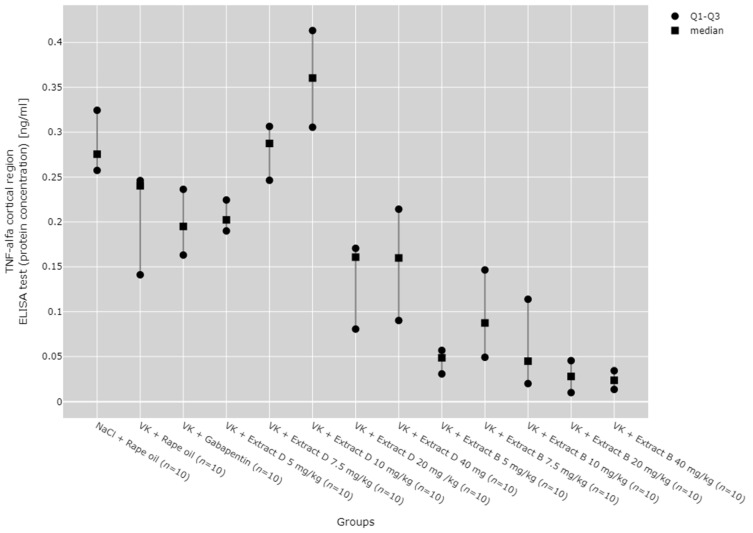
Comparison of TNF-α protein concentrations in the cortical region between groups, measured by ELISA assay. Statistical analysis was performed using the Kruskal–Wallis test (*p* < 0.001). Post hoc test results: VK + Extract B 5 mg/kg vs. NaCl + Rape Oil (*p* = 0.005), VK + Extract B 5 mg/kg vs. VK + Extract D 7.5 mg/kg (*p* = 0.008), VK + Extract B 5 mg/kg vs. VK + Extract D 10 mg/kg (*p* < 0.001), VK + Extract B 10 mg/kg vs. NaCl + Rape Oil (*p* = 0.001), VK + Extract B 20 mg/kg vs. VK + Rape Oil (*p* = 0.044), VK + Extract B 10 mg/kg vs. VK + gabapentin (*p* = 0.048), VK + Extract B 10 mg/kg vs. VK + Extract D 7.5 mg/kg (*p* = 0.002), VK + Extract B 10 mg/kg vs. VK + Extract D 10 mg/kg (*p* < 0.001), VK + Extract B 40 mg/kg vs. NaCl + Rape Oil (*p* < 0.001), VK + Extract B 40 mg/kg vs. VK + Rape Oil (*p* = 0.012), VK + Extract B 40 mg/kg vs. VK + gabapentin (*p* = 0.012), VK + Extract B 40 mg/kg vs. VK + Extract D 7.5 mg/kg (*p* < 0.001), VK + Extract B 40 mg/kg vs. VK + Extract D 10 mg/kg (*p* < 0.001) (only statistically significant results are presented).

**Figure 16:**
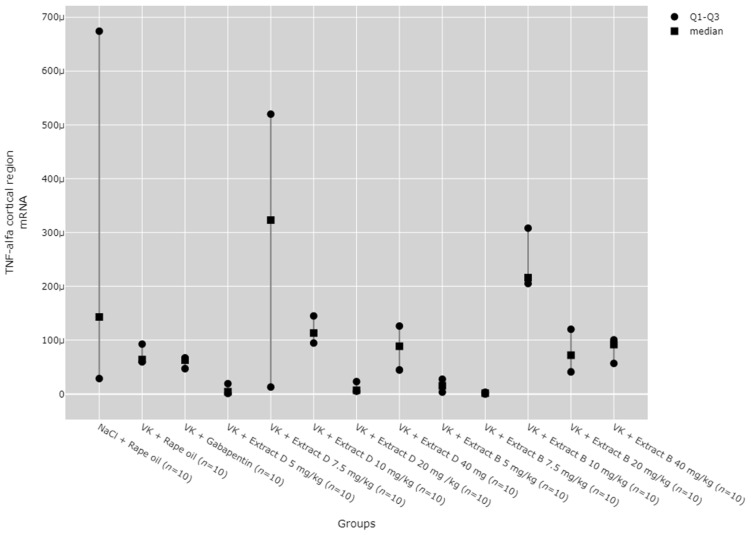
Comparison of TNF-α mRNA expression in the cortical region between groups. Statistical analysis was performed using the Kruskal–Wallis test (*p* < 0.001). Post hoc test results: Extract B 10 mg/kg vs. Extract D 5 mg/kg (*p* = 0.002), Extract B 10 mg/kg vs. Extract B 5 mg/kg (*p* = 0.014), Extract B 10 mg/kg vs. Extract B 7.5 mg/kg (*p* = 0.006) (only statistically significant results are presented).

**Figure 17:**
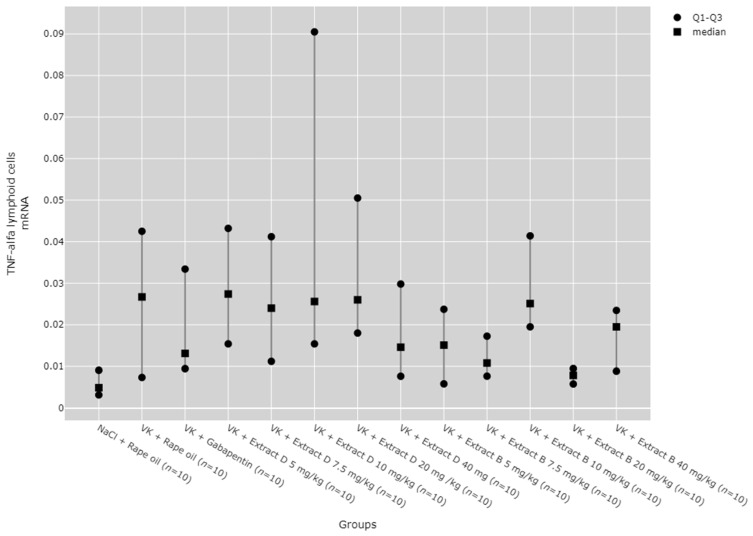
Comparison of TNF-α mRNA expression in the lymphoid cells between groups. Statistical analysis was performed using the Kruskal–Wallis test (*p* = 0.033).

There were statistically significant differences between all TNF-α mRNA and protein levels in the cortex (*p* < 0.001), hippocampus (*p* < 0.001), and lymphocytes (*p* = 0.033, Figure 13, Figure 14, Figure 15, Figure 16 and Figure 17).

Post hoc test results showed that in the hippocampus, TNF-α protein expression increased after administering 7.5 mg/kg of Extract B (VK + Extract B 7.5 mg/kg) compared to the NaCl group (NaCl + Rape Oil, *p* = 0.004), the vincristine group (VK + Rape Oil, *p* = 0.006), the doses of 5 mg/kg (*p* = 0.006), 7.5 mg/kg (*p* = 0.025), 10 mg/kg (*p* = 0.017), and 20 mg/kg (*p* = 0.001) of Extract D (VK +Extract D), and the 5 mg/kg dose of Extract B (VK + Extract B 5 mg/kg, *p* = 0.004, Figure 13).

The administration of 7.5 mg/kg of Extract B (Extract B 7.5 mg/kg) resulted in a statistically significant elevation in TNF-α mRNA levels in the hippocampus when compared to the NaCl control group (NaCl + Rape Oil, *p* = 0.002), the gabapentin-treated group (VK + gabapentin, *p* = 0.001), and the group treated with 7.5 mg/kg of Extract D (VK + Extract D 7.5 mg/kg, *p* = 0.014). Likewise, the 10 mg/kg dose of Extract B (VK + Extract B 10 mg/kg) also led to a significant increase in TNF-α mRNA expression relative to the NaCl group (NaCl + Rape Oil, *p* = 0.026) and the gabapentin group (VK + gabapentin, *p* = 0.008; Figure 14).

In the cerebral cortex, the 5 mg/kg dose of Extract B (VK + Extract B 5 mg/kg) reduced TNF-α protein expression in comparison to the NaCl group (NaCl + Rape Oil, *p* = 0.005), the 7.5 mg/kg dose of Extract D (Extract D 7.5 mg/kg, *p* = 0.008), and the 10 mg/kg dose of Extract D (Extract D 10 mg/kg, *p* < 0.001).

The 20 mg/kg dose of Extract B (VK + Extract B 20 mg/kg) similarly decreased TNF-α protein levels in the cortex compared to the NaCl group (NaCl + Rape Oil, *p* = 0.001), the vincristine group (VK + Rape Oil, *p* = 0.044), the gabapentin group (VK + gabapentin, *p* = 0.048), and both the 7.5 mg/kg and 10 mg/kg doses of Extract D (Extract D 7.5 mg/kg, *p* = 0.002; Extract D 10 mg/kg, *p* < 0.001). The 40 mg/kg dose of Extract B (VK + Extract B 40 mg/kg) further decreased TNF-α protein levels in the cortex relative to the NaCl group (NaCl + Rape Oil, *p* < 0.001), the vincristine group (VK + Rape Oil, *p* = 0.012), the gabapentin group (VK + gabapentin, *p* = 0.012), and the 7.5 mg/kg and 10 mg/kg doses of Extract D (Extract D 7.5 mg/kg, *p* < 0.001; Extract D 10 mg/kg, *p* < 0.001; Figure 15).

Finally, the 10 mg/kg dose of Extract B (Extract B 10 mg/kg) led to an increase in TNF-α mRNA expression in the cerebral cortex compared to the 5 mg/kg dose of Extract D (Extract D 5 mg/kg, *p* = 0.002), as well as the 5 mg/kg and 7.5 mg/kg doses of Extract B (Extract B 5 mg/kg, *p* = 0.014; Extract B 7.5 mg/kg, *p* = 0.006; Figure 16).

Although statistically significant differences between groups were detected for TNF-α mRNA expression in lymphoid cells (*p* = 0.033), no significant results were observed in the post hoc tests (Figure 17).

No significant dose-dependent trends were observed for TNF-α protein or mRNA expression in the hippocampus (Figure 13 and Figure 14). Trend analysis for TNF-α protein expression showed a statistically significant dose-dependent decrease in the range of 7.5 mg/kg to 40 mg/kg for Extract B in the cerebral cortex (*p* = 0.011, Figure 15). For both extracts, no significant dose-dependent trends were observed at the mRNA level for TNF-α in lymphocytes (Extract B: *p* = 0.310, Extract D: *p* = 0.334, Figure 17).

### 2.6. NFkB mRNA Expression in the Rat Brain and Blood Lymphocytes

**Figure 18:**
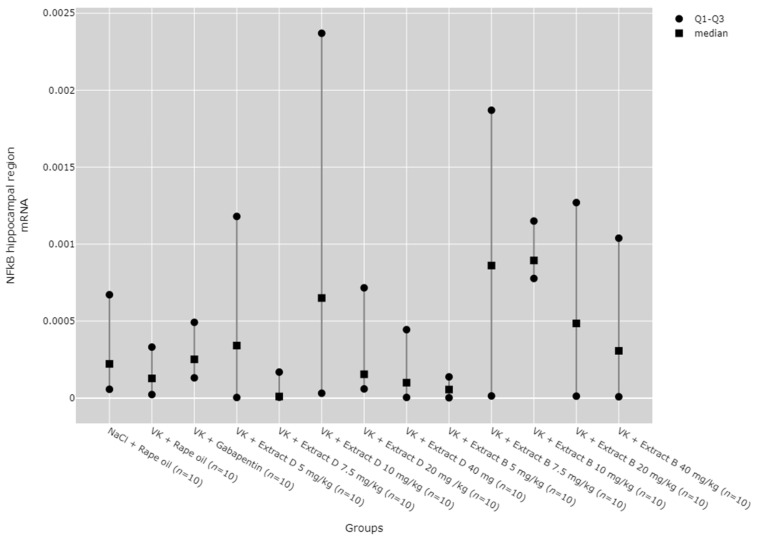
Comparison of NF-κB mRNA expression in the hippocampal region between groups. Statistical analysis was performed using the Kruskal–Wallis test (*p* = 0.157).

**Figure 19:**
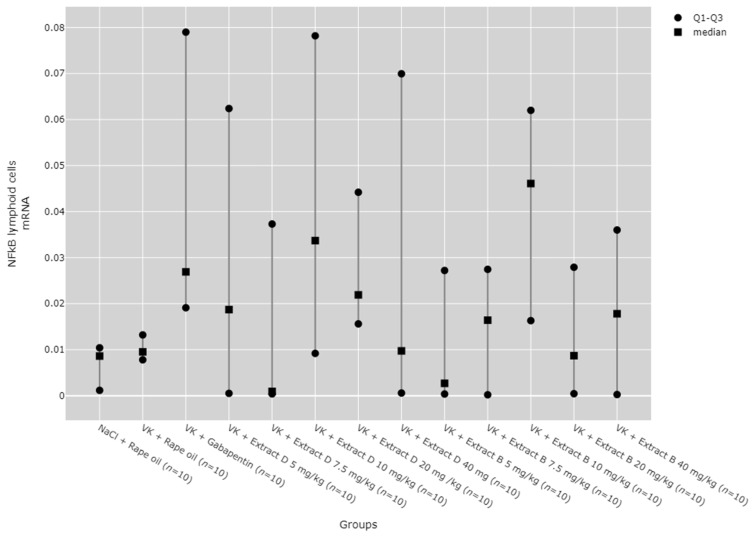
Comparison of NF-κB mRNA expression in the lymphoid cells between groups. Statistical analysis was performed using the Kruskal–Wallis test (*p* = 0.428).

**Figure 20:**
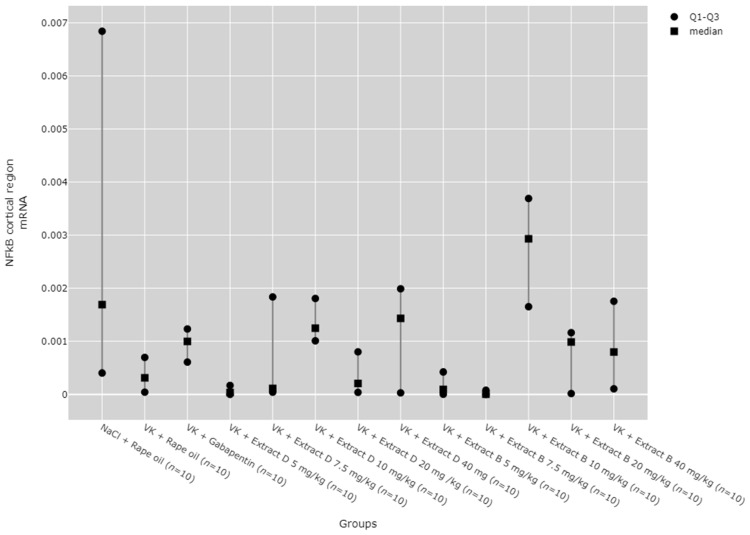
Comparison of NF-κB mRNA expression in the cortical region between groups. Statistical analysis was performed using the Kruskal–Wallis test (*p* = 0.026).

There were no statistically significant differences between groups in NF-κB mRNA expression in the hippocampus (Figure 18) and lymphocytes (Figure 19). While significant differences between groups were detected for NF-κB mRNA expression in the cortical region using the Kruskal–Wallis test, no significant pairwise differences were identified in the post hoc tests (Figure 20). Furthermore, trend analysis indicated no significant changes in NF-κB mRNA expression in relation to the dose-dependent administration of both extracts in all tissues assessed.

## 3. Discussion

Pharmacotherapy for neuropathic pain remains a major challenge as currently used drugs often provide only partial or short-term improvement [19]. Various drugs are used depending on the underlying cause of pain symptoms, such as tricyclic antidepressants, serotonin and norepinephrine reuptake inhibitors, α2-δ subunit agonists of the Ca^2+^ channel, lidocaine, and opioids. Combined therapy may be more effective than monotherapy, but the selection of appropriate pharmacological treatment should also consider other coexisting diseases [20]. Natural cannabinoids have emerged as a promising group of compounds in pain therapy, including neuropathic pain [21]; therefore, the present study was designed to evaluate the impact of cannabinoids derived from *Cannabis sativa* on the inflammatory process in the brain (hippocampus and cerebral cortex) and peripherally in lymphocytes of rats with vincristine-induced neuropathic pain. We analyzed extracts of *Cannabis sativa* from two distinct plant varieties, significantly extending the current body of knowledge, particularly in the context of chemotype diversity and their pharmacological effects. Unlike many studies that analyzed a single product derived from one cannabis strain, which precludes comparison across different varieties [22], our findings consider the biological diversity of plants, allowing us to observe variations in the expression of inflammatory factors in response to different extracts. Furthermore, our previous studies demonstrated the modulation of cannabinoid receptor expression in both the brain and peripheral blood lymphocytes, supporting the notion that cannabis extracts may exert location-specific effects [23].

Our findings, combined with observations from other authors, emphasize that the full spectrum of extract components, including terpenes and other bioactive compounds, plays a crucial role in their pharmacological activity [24,25]. We observed that the chemical composition diversity of the studied cannabis varieties influences differences in their effects. This observation aligns with other studies highlighting the significance of terpenes such as β-myrcene, α-pinene, and β-caryophyllene, which exhibit anti-inflammatory and analgesic properties [26,27].

The molecular background of neuropathic pain is discussed and many hypotheses have been presented. One suggests that the inflammatory microenvironment and the release of pro-inflammatory mediators, like cytokines, rather than nerve injury, are pivotal for developing neuropathic pain [28]. Cytokines play a pivotal role in the development of neuropathy by increasing the excitability of sensory neurons through the activation of sodium and calcium channels, leading to hyperalgesia and allodynia, thereby confirming the involvement of inflammation in the pathophysiology of neuropathic pain [29].

Therefore, according to the above hypothesis, inflammation accompanies neuropathic pain development and transduction by the accumulation and recruitment of inflammatory factors.

Our analysis revealed significant differences in the expression of inflammatory markers (COX-1, COX-2, TNF-α) in various brain regions, especially in the hippocampus and cerebral cortex, suggesting that CSL Extracts D and B significantly influence COX-1, COX-2, and TNF-α expression in both brain regions, with dose-dependence and efficacy differences between the two areas (Supplementary Materials).

Despite the available literature confirming the anti-inflammatory properties of cannabinoids [30,31], the present study demonstrated that CSL extracts may exert complex and region-specific effects on inflammatory processes in the brain and peripherally. The effects of the extracts on inflammatory processes in the brain are not only tissue-specific (hippocampus vs. cerebral cortex vs. lymphocytes) but also dependent on the type of extract used. Extracts B and D differ in terms of the intensity and nature of their modulation of inflammation. Extract B exerted a pronounced pro-inflammatory effect in the hippocampus, significantly increasing COX-2 and TNF-α protein expression, whereas Extract D induced a moderate inflammatory response in this region, primarily affecting COX-1 expression. In contrast, Extract B reduced COX-2 and TNF-α expression in the cerebral cortex, suggesting its potential anti-inflammatory action in this tissue. Clinical observations confirmed that cyclooxygenases and TNF-α participate in the peripheral mediation of neuropathic pain during chemotherapy, which produces peripheral neuropathy with massive release of this factor in serum [32]. Extract D, however, caused a dose-dependent increase in COX-1, but its influence on other inflammatory markers appeared less pronounced. The two extracts also differed in their effects on the same inflammatory pathways. Extract B exhibited a more complex and intense influence, particularly on pathways related to COX-2 and TNF-α, making it more active in modulating inflammatory processes, whereas Extract D appeared to act more steadily and predictably in regulating COX-1, especially in a dose-dependent manner. However, the absence of significant differences in NF-κB mRNA expression in the analyzed tissues suggests that these CSL extracts do not affect the activation of NF-κB-related signalling pathways, at least within the dosage range studied. All trend analyses indicate a lack of significant changes in NF-κB mRNA expression in the context of dose-dependency, which may confirm that their effects are not associated with this particular signalling pathway. Our results on variable inflammatory factor expression are partly consistent with the observations of other authors, who demonstrated that CBD acts through various mechanisms, including the modulation of serotonergic receptors (5-HT1A), TRPV1 channels, and GABA receptors [33,34,35].

This complexity likely originates from the rich and varied composition of CSL extracts which contain more than 400 compounds, of which only a select few, such as THC and CBD, are well understood. Notably, some compounds selectively target CB1 and CB2 receptors, thereby modulating inflammatory processes through either inhibition or activation [36].

CB1R and CB2R receptors play a key role in the analgesic and anti-inflammatory effects of cannabinoids. Interestingly, the analgesic effects of CBD are not always dependent on CB1 and CB2 receptors, as also confirmed in earlier studies [37]. In our previous study on cannabinoid receptors [19], we demonstrated that vincristine induced a decrease in CB1R and CB2R expression in the cerebral cortex, while gabapentin increased CB1R levels in the hippocampus. These findings suggest that the synergistic action of CBD with other compounds present in the extract might influence their pharmacological activity differently depending on the cannabis variety used. Moreover, our results suggest that pain modulation via CB1R and CB2R may be brain region-specific, consistent with the observed changes in inflammatory mediators. Extract B increased CB1R expression at the protein level in the hippocampus but decreased it in the cortex at higher doses (10–40 mg/kg). This differential regulation indicates that the role of CB1R may be closely tied to local inflammatory processes, such as those mediated by COX-2 and TNF-α, which also showed distinct expression patterns in these regions. The increased expression of COX-2 and TNF-α in the hippocampus could be associated with the rise in CB1R levels, potentially amplifying inflammatory pathways. CB2R is primarily linked to immune system modulation. The observed increase in CB2R expression, particularly following the administration of Extract B, correlated with reduced COX-2 response in certain brain areas, suggesting that CB2R activation may counterbalance pro-inflammatory signals like COX-2 and TNF-α in a dose-dependent manner. Regarding immunomodulation, our results complement the insights of other researchers who highlight the immunosuppressive properties of CBD and selected terpenes, such as α-pinene [38]. Our data show that the tested cannabis varieties differentially modulate the expression of inflammatory factors, further affirming the importance of the chemical diversity of extracts.

The present study has some limitations. Interpreting results related to plant extracts requires careful consideration as they contain a complex array of compounds beyond the primary active substances (e.g., CBD, THC), which may simultaneously affect multiple signalling pathways and produce pleiotropic effects. Nevertheless, the analysis of extracts is essential as it reflects the interactions between the full spectrum of compounds present in *Cannabis sativa* leaves. It is important to note that only gene expression in lymphocytes due to limited material available for protein isolation. Also, no Bonferroni correction was applied for multiple comparisons, which may have influenced the interpretation of results.

## 4. Materials and Methods

### 4.1. Cannabis sativa *L.* (CSL) Extract Preparation

Extracts were prepared from two *Cannabis sativa* varieties, KC Dora, a Hungarian variety designated as Hemp flower extract version D and Tygra, designated as Hemp flower extract version T, derived from a Polish strain. The extracts were characterized using HPLC according to the methodology outlined by Zielinska et al. [18], and the composition of the KC Dora and Tygra varieties, which include a Hungarian fibrous monoecious hemp variety bred at Agromag Kft (referred to as Hemp Flower Extract version D) and a Polish strain of industrial monoecious hemp cultivated at the Institute of Natural Fibres and Medicinal Plants (designated as Hemp Flower Extract version B), was published in an earlier publication [17,23]. Extract B was prepared from the *Cannabis sativa* L. Tygra variety. Female inflorescences, known for their high cannabinoid content, were collected and dried. The dried material was crumbled to separate stems and seeds, then sieved through a 0.63 mm mesh to obtain a fine powder. For the extraction, 10 g of this powder was transferred to a 250 mL flask, and 100 mL of an ethanol–water mixture (20%, 40%, or 80% ethanol) was added. The flask was covered with aluminum foil to protect the sample from light. The mixture was subjected to maceration by stirring it at room temperature for three days at a constant speed of 100 rpm. After the maceration process, the extract was filtered through double-folded gauze to remove solid residues, and the filtrate was collected for further analysis.

Extract D was derived from the *Cannabis sativa* L. Dora variety using ultrasound-assisted extraction. Like for Extract B, female inflorescences were harvested, dried, and processed into a fine powder by crumbling and sieving through a 0.63 mm mesh. For this method, 2 g of the prepared powder was placed in a 250 mL flask, and 10 mL of 96% ethanol was added. The flask was then subjected to ultrasonic treatment in an ultrasound water bath operating at a frequency of 40 kHz for 30 min. After sonication, the extract was filtered using double-folded gauze to remove solid residues, and the filtrate was collected.

Both extracts underwent subsequent analysis using High-Performance Liquid Chromatography (HPLC). The separation of cannabinoids (CBD, CBDA, Δ9-THC, and Δ9-THCA) was performed on an Accucore C18 column under controlled conditions. The cannabinoids were identified by comparing retention times with standard compounds, and quantification was achieved using calibration curves prepared for each compound. This analytical process ensured precise measurement of the cannabinoid content in both extracts [17,18].

### 4.2. Animal Study

Male animals (Wistar rats with a body weight of 200–250 g) were housed in groups of five per cage at a temperature of 22 ± 2 °C, with a 12 h light/dark cycle, and had unrestricted access to food and water for at least one week prior to the experiment. The rats used in the study were aged 7–8 weeks old. Neuropathic pain was induced by intraperitoneal (i.p.) vincristine administration at a dose of 0.1 mg/kg body weight (bw) for five consecutive days, followed by two days of saline solution in the same volume. This treatment cycle was repeated twice, after which gabapentin (60 mg/kg bw) was administered alone or in combination with CSL extracts and CBD via oral gavage for five days. Doses were calculated based on their cannabidiol content (5, 7.5, 10, 20, and 40 mg/kg), and the characteristics of the treatment groups are outlined in Table 1 [23].

Neuropathic pain was confirmed by the tail-flick and von Frey tests as described previously [19]. The rats were euthanized by decapitation to obtain brain tissue and blood samples. Lymphocytes were separated from the peripheral blood using Gradisol, while total RNA was extracted from the brain tissue (frontal cortex, hippocampus) and blood lymphocytes using TriPure Isolation Reagent (Roche, Roche Diagnostics Polska Sp. z o.o., Warszawa, Poland), in accordance with the manufacturer’s instructions [39].

Pharmacological experiments were carried out in collaboration with the Department of Pharmacology at the University of Medical Sciences in Poznan. The study protocol received approval from the local Ethics Committee for Animal Research (Resolution No. 42/2015) and was conducted following the relevant guidelines for laboratory animal care and ethical standards for pain research in conscious animals.

### 4.3. Real-Time PCR

Approximately 1–2 μg of total RNA was reverse transcribed using the Transcriptor First Strand cDNA Synthesis Kit (Roche) according to the manufacturer’s protocol. The expression levels of COX-1, COX-2, and TNF-α mRNA in the hippocampus, frontal cortex, and peripheral blood lymphocytes were analyzed using real-time PCR. The LightCycler TM system (Roche, Penzberg, Germany) and the LightCycler Fast Start DNA Master SYBR Green I kit (Roche Applied Science, Germany) were employed for amplification. GAPDH served as the housekeeping gene (internal control) for normalization. The sequences of the primers used in the assay are listed in Table 2.

### 4.4. ELISA

The levels of COX-1, COX-2, and TNF-α proteins were measured in rat tissue homogenates, cell lysates, and other biological fluids using an ELISA method (ELISA procedures were conducted in accordance with the manufacturer’s guidelines for ELISA kits, including Cannabinoid Receptor 1 (CNR1), Cannabinoid Receptor 2 (CNR2), Prostaglandin Endoperoxide Synthases 1 and 2 (PTGS1 and PTGS2), Tumour Necrosis Factor Alpha (TNFa), Glutamate Receptor, Ionotropic, N-Methyl-D-Aspartate 1 (GRIN1), and Glutamate Receptor, Ionotropic, N-Methyl-D-Aspartate 2A (GRIN2A), and Nuclear Factor Kappa B (NFkB). These kits were employed following their specific instructions to measure relevant biomarkers). To prepare the tissue homogenates, tissue samples were first washed in ice-cold PBS (0.01 mol/L, pH 7.0–7.2) to eliminate residual blood, followed by homogenization in 5–10 mL of PBS with a glass homogenizer on ice. The resulting homogenates were centrifuged at 5000× *g* for 5 min, and the total protein concentration was determined using the BCA (bicinchoninic acid) assay. The homogenate samples, along with the COX-1, COX-2, and TNF-α standards, were then placed in a 96-well plate to allow binding to the immobilized antibodies. Absorbance was measured to create a standard curve, enabling the quantification of COX-1, COX-2, and TNF-α concentrations.

### 4.5. Statistical Analysis

Statistical analysis was performed using the Statistica 13 software (TIBCO Software Inc., Palo Alto, CA, USA) and the PQStat 1.8.4 software (PQStat Software, Poznań/Plewiska, Poland). A significance level of *p* < 0.05 was adopted. The normality of the variable distributions was assessed using the Shapiro–Wilk test. Because many variables did not follow a normal distribution, the Kruskal–Wallis test followed by Dunn’s post hoc test was employed to compare groups. The Jonckheere–Terpstra trend test was applied separately for each extract to compare the effects of different doses. Data visualization was performed using the Python programming language and the Plotly library. The figures present median values along with interquartile ranges (IQRs).

## 5. Conclusions

*Cannabis sativa* L. extracts significantly influenced the expression of inflammatory genes and proteins, with effects varying based on dose and tissue type.

The increased expression of COX-1, COX-2, and TNF-α in the hippocampus and COX-1 in the cerebral cortex suggests that *Cannabis* may have a pro-inflammatory effect.

Due to species specificity, the results of our research based on rats require confirmation in humans. However, *Cannabis sativa* should be recommended with caution for treating pain with an inflammatory component.

## Figures and Tables

**Table 1:** Treatment group characteristics.

Category	Substance Administered	Animals (n)
1	NaCl (0.9%, 1 mL i.p.) + Rape Oil (1 mL p.o.)	10
2	VK + Rape Oil—1 mL p.o.	10
3	VK + gabapentin (solution 1 mg/mL)—60 mg/kg bw p.o.	10
4	VK + Extract D—5.0 mg/kg bw p.o	10
5	VK + Extract D—7.5 mg/kg bw p.o	10
6	VK + Extract D—10.0 mg/kg bw p.o	10
7	VK + Extract D—20.0 mg/kg bw p.o	10
8	VK + Extract D—40.0 mg/kg bw p.o	10
9	VK + Extract B—5.0 mg/kg bw p.o	10
10	VK + Extract B—7.5 mg/kg bw p.o	10
11	VK + Extract B—10.0 mg/kg bw p.o	10
12	VK + Extract B—20.0 mg/kg bw p.o	10
13	VK + Extract B—40.0 mg/kg bw p.o	10

Extract B—*Cannabis sativa* L. extract, variety Tygra, administered orally, with the dose expressed as synthetic cannabidiol. Extract D—*Cannabis sativa* L. extract, variety Dora, administered orally, with the dose expressed as synthetic cannabidiol. VK—vincristine sulphate solution (1 mg/mL), injected intraperitoneally (i.p.) at a dosage of 0.1 mg/kg body weight (bw). i.p.—intraperitoneal; p.o.—oral administration; bw—body weight.

**Table 2:** Primer sequences.

Gene	Sequence 5′ → 3′
COX-1	Primer-F: 5′ TCTATGCTGGTGGACTACG 3′ Primer-R: 5′ CATCTCCTTCTCTCCTGTG 3′
COX-2	Primer F: 5′ CTACGCCTGAGTTTCTGAC 3′ Primer R: 5′ ATTGTAAGTTGGTGGGCTG 3′
TNF-α	Primer F: GCTCCCTCTCATCAGTTCC Primer R: GCTTGGTGGTTTGCTACG
NFkB	Primer F: ACACCTCTACACATAGCAG Primer R: CTACTCCCTCATCTTCTCC
GADPH	Primer-F.: 5′ GATGGTGAAGGTCGGTGTG 3 Primer-R.: 5′ ATGAAGGGGTCGTTGATGG 3′

## Data Availability

Data are contained within the article and Supplementary Materials.

## Supplementary Materials

The following supporting information can be downloaded at: https://www.mdpi.com/article/10.3390/molecules30010194/s1, Table S1. COX-1 receptor expression in the brain cortex, hippocampus and lymphocytes in response to treatment with vincristine, gabapentin and CSL extracts. Table S2. COX-2 expression in the brain cortex, hippocampus and lymphocytes in response to treatment with vincristine, gabapentin and CSL extracts. Table S3. TNF expression in the brain cortex, hippocampus and lymphocytes in response to treatment with vincristine, gabapentin and CSL extracts.

## List of Abbreviations

**Abbreviation**	**Definition**
CBD	Cannabidiol
CBD-A	Cannabinoid acid
CB1R	Cannabinoid receptor 1
CB2R	Cannabinoid receptor 2
CNR1	Cannabinoid receptor 1 gene
CNR2	Cannabinoid receptor 2 gene
COX-1	Cyclooxygenase-1
COX-2	Cyclooxygenase-2
Δ9-THC	THC-Δ9-Tetrahydrocannabinol, Tetrahydrocannabinol
Δ9-THC-A	Δ9-Tetrahydrocannabinoid acid
ELISA	Enzyme-Linked Immunosorbent Assay
Extract B	*Cannabis sativa* L. extract from the Tygra variety, administered orally as synthetic cannabidiol
Extract D	*Cannabis sativa* L. extract from the Dora variety, administered orally as synthetic cannabidiol
i.p.	Intraperitoneal administration
NFκB	Nuclear Factor kappa B
p.o.	Oral administration (per os)
RT-PCR	Real-Time Polymerase Chain Reaction
TNFα	Tumour Necrosis Factor alpha
Vin	Vincristine administered at 0.1 mg/kg body weight, via intraperitoneal injection
bw	Body weight
