# Supplementary material for: Cannabis sativa L. Extract Increases COX-1, COX-2 and TNF-α in the Hippocampus of Rats with Neuropathic Pain
Source: Molecules. 2025 Jan 6;30(1):194. doi: 10.3390/molecules30010194 (PMC11721898; doi:10.3390/molecules30010194)
Supplement: Supplementary file 1 [file molecules-30-00194-s001.zip › molecules-3342205-supplementary.pdf]

Supplement

Cannabis Sativa L. Extract Increases COX-1, COX-2 and TNF-α in the Hippocampus of Rats with Neuropathic Pain

Joanna Bartkowiak-Wieczorek [1,*], Małgorzata Jamka [2], Radosław Kujawski [3], Marcin Hołysz [4], Agnieszka Bienert [3], Kamila Czora-Poczwardowska [3], Michał Szulc [3], Przemysław Mikołajczak [3], Anna Bogacz [1], Anna-Maria Wizner [5], Karolina Wielgus [2], Ryszard Słomski [6] and Edyta Mądry [1]

[1] Physiology Department, Poznan University of Medical Sciences, 6, Święcickiego Street, 60-781 Poznan, Poland; aniabogacz23@o2.pl (A.B.); emadry@ump.edu.pl (E.M.)
[2] Department of Paediatric Gastroenterology and Metabolic Diseases, Poznan University of Medical Sciences, Szpitalna Street 27/33, 60-572 Poznan, Poland; mjamka@ump.edu.pl (M.J.); kwielgus@ump.edu.pl (K.W.)
[3] Department of Pharmacology, Poznan University of Medical Sciences, 3, Rokietnicka Street,
60-806 Poznan, Poland; radkuj@ump.edu.pl (R.K.); agbienert@ump.edu.pl (A.B.); kczora@ump.edu.pl (K.C.-P.); mszulc@ump.edu.pl (M.S.); przemmik@ump.edu.pl (P.M.)
[4] Department of Biochemistry and Molecular Biology, Poznan University of Medical Sciences, 6, Swiecickiego Steet, 60-781 Poznan, Poland; mholysz@ump.edu.pl
[5] Department of Clinical Pharmacy and Biopharmacy, Poznan University of Medical Sciences, 3, Rokietnicka Street, 60-806 Poznan, Poland; anna.usciniak@gmail.com
[6] Department of Biotechnology, Institute of Natural Fibres and Medicinal Plants—National Research
Institute, Wojska Polskiego 71B, 60-630 Poznan, Poland; slomski@up.poznan.pl
* Correspondence: joannawieczorek@ump.edu.pl

RESULTS

Table S1. COX-1 receptor expression in the brain cortex, hippocampus and lymphocytes in response to treatment with vincristine, gabapentin and CSL extracts

COX-1					
Treatment Group	Hippocampus		Cortex		Lymphocytes
	Protein [ng/ml] Median (1st; 3rd quartile)	Gene expression Median (1st; 3rd quartile)	Protein [ng/ml] Median (1st; 3rd quartile)	Gene expression Median (1st; 3rd quartile)	Gene Expression Median (1st; 3rd quartile)
NaCl- 1ml i.p. + Rape oil -1 ml p.o.	7.15 (5.64; 7.77)	1.91E-04 (6.10E-05; 1.10E-03)	10.95 (7.753; 12.71)	1.89E-03 (3.46E-04; 7.00E-03)	1.55E-02 (9.77E-04; 1.48E-01)
VK +Rape oil	4.45 (4.32; 5.12)	3.84E-04 (2.25E-04; 9.46E-04)	19.02 (12.59; 25.74)	9.36E-04 (7.25E-04; 1.07E-03)	2.67E-02 (7.37E-03; 4.25E-02)
VK + Gabapentin	5.77 (4.23; 6.1)	1.59E-04 (1.29E-04; 3.50E-04)	7.26 (4.93; 10.9)	8.26E-04 (6.78E-04; 1.12E-03)	1.43E-01 (8.54E-02; 1.61E-01)
VK + Extract D 5 mg/kg	3.23 (2.47; 3.71)	4.63E-04 (3.47E-04; 1.19E-03)	6.75 (4.53; 15.21)	1.60E-05 (6.00E-06; 3.40E-05)	1.59E-01 (1.18E-01; 1.78E-01)
VK + Extract D 7.5 mg/kg	11.8 (7.9; 13.8)	3.57E-04 (2.96E-04; 6.69E-04)	11.4 (10.84; 17)	6.99E-04 (6.00E-06; 6.11E-03)	1.91E-01 (1.07E-01; 2.52E-01)
VK + Extract D 10 mg/kg	12.47 (10.11; 18.13)	2.31E-03 (1.99E-03; 3.97E-03)	15.06 (10.64; 17.68)	1.33E-03 (9.82E-04; 1.97E-03)	5.64E-02 (2.38E-02; 1.06E-01)
VK + Extract D 20 mg /kg	22.48 (21.99; 29.81)	8.98E-04 (5.40E-05; 1.40E-03)	17.67 (15.67; 25.28)	2.49E-04 (5.60E-05; 4.72E-04)	6.62E-02 (4.74E-02; 2.18E-01)
VK + Extract D 40 mg	8.21 (6.65; 10.03)	4.79E-04 (1.39E-04; 1.08E-03)	17.90 (15.26; 20.44)	1.55E-03 (1.01E-03; 2.00E-03)	1.68E-01 (2.82E-02; 1.96E-01)
VK + Extract B 5 mg/kg	5.92 (5.15; 11.46)	3.59E-04 (1.04E-04; 9.37E-04)	15.11 (12.81; 21.29)	2.90E-04 (5.70E-05; 5.25E-04)	1.11E-01 (7.57E-02; 1.77E-01)
VK + Extract B 7.5 mg/kg	11.55 (6.27; 18.74)	1.86E-03 (1.51E-03; 2.62E-03)	18.77 (16.17; 21.88)	3.00E-06 (0.00E+00; 9.60E-05)	1.08E-01 (1.44E-02; 1.98E-01)
VK + Extract B 10 mg/kg	14.70 (12.60; 20.00)	3.79E-03 (3.45E-03; 5.13E-03)	19.60 (17.20; 25.00)	3.35E-03 (3.22E-03; 3.48E-03)	5.45E-02 (1.72E-02; 2.34E-01)
VK + Extract B 20 mg/kg	10.59 (6.22; 13.80)	2.34E-03 (2.25E-03; 2.65E-03)	13.57 (11.38; 14.90)	3.75E-03 (2.99E-03; 4.24E-03)	3.94E-02 (2.33E-02; 6.40E-02)
VK + Extract B 40 mg/kg	11.71 (11.10; 14.67)	1.11E-03 (7.10E-04; 1.36E-03)	22.06 (20.27; 25.95)	3.35E-03 (2.06E-03; 4.13E-03)	1.22E-01 (9.09E-02; 1.58E-01)
P (Kruskal-Wallis)	p<0.001[1]	p<0.001[2]	p<0.001[3]	p<0.001[4]	p=0.09

[1] VK+ Extract D 5 mg/kg *vs* VK+ Extract D 7.5 mg/kg (p=0.026); VK+ Extract D 5 mg/kg *vs* VK+ Extract D 10 mg/kg (p=0.012); VK+ Extract D 5 mg/kg *vs* VK+ Extract D 20 mg/kg (p<0.001); VK+ Extract D 5 mg/kg *vs* VK+Extract B 7.5 mg/kg (p=0.003); VK+ Extract D 5 mg/kg *vs* VK+Extract B 10 mg/kg (p<0.001); VK+ Extract D 5 mg/kg *vs* VK+Extract B 40 mg/kg (p=0.001); VK+ Extract D 20 mg/kg vs NaCl- 1ml+ Rape oil -1 ml (p=0.021); VK+ Extract D 20 mg/kg vs VK+Rape oil (p<0.001); VK+ Extract D 20 mg/kg vs VK+gabapentin (p=0.002); VK+Extract B 10 mg/kg vs VK+Rape oil (p=0.004); VK+Extract B 10 mg/kg vs VK+gabapentin (p=0.019)
[2] VK+ Extract D 10 mg/kg *vs* NaCl- 1ml+ Rape oil -1 ml (p=0.048); VK+ Extract D 10 mg/kg *vs* VK+gabapentin (p=0.006); VK+Extract B 7.5 mg/kg *vs* VK+gabapentin (p=0.009); VK+Extract B 10 mg/kg *vs* NaCl- 1ml+ Rape oil -1 ml (p=0.001); VK+Extract B 10 mg/kg *vs* VK+Rape oil (p=0.010); VK+Extract B 10 mg/kg *vs* VK+gabapentin (p<0.001); VK+Extract B 10 mg/kg *vs* VK+Extract D 5 mg/kg (p=0.035); VK+Extract B 10 mg/kg *vs* VK+Extract D 7.5 mg/kg (p=0.004); VK+Extract B 10 mg/kg *vs* VK+ Extract D 20 mg/kg (p=0.027); Extract B 10 mg/kg *vs* Extract D 40 mg/kg (p=0.003); VK+Extract B 10 mg/kg *vs* VK+Extract B 5 mg/kg (p=0.002); VK+ Extract D 20 mg/kg *vs* NaCl- 1ml+ Rape oil -1 ml (p=0.034); VK+ Extract D 20 mg/kg *vs* VK+gabapentin (p=0.004)
[3] VK+ Extract B 40 mg/kg *vs* NaCl- 1ml+ Rape oil -1 ml (p=0.008); VK+ Extract B 40 mg/kg *vs* VK+gabapentin (p=0.001); VK+ Extract B 40 mg/kg *vs* Extract D 5 mg/kg (p=0.002)
[4] VK+ Extract B 10 mg/kg *vs* VK+ Extract D 5 mg/kg (p=0.001); VK+ Extract B 10 mg/kg *vs* VK+ Extract B 7.5 mg/kg (p=0.018); VK+ Extract B 20 mg/kg *vs* VK+ Extract D 5 mg/kg (p=0.001); VK+ Extract B 20 mg/kg *vs* VK+ Extract B 7.5 mg/kg (p=0.0132); VK+ Extract B 40 mg/kg *vs* VK+ Extract D 5 mg/kg (p=0.011)

RT-PCR-Real Time PCR ELISA- enzyme-linked immunosorbent assay Vin- Vincristine - 0.1 mg/kg of body weight, intraperitoneal administration Extract D - *Cannabis sativa L extract, variety Dora*; the dose expressed as synthetic Cannabidiol, oral administration Extract B - *Cannabis sativa L extract, variety Tygra;* the dose expressed as synthetic Cannabidiol, oral administration i.p.– intraperitoneal; p.o.– per os; bw – body weight.

Table S2. COX-2 expression in the brain cortex, hippocampus and lymphocytes in response to treatment with vincristine, gabapentin and CSL extracts

COX-2					
**Treatment Group**	**Hippocampus**		**Cortex**		**Lymphocytes**
	Protein [ng/ml] Median (1st; 3rd quartile)	Gene expression Median (1st; 3rd quartile)	Protein [ng/ml] Median (1st; 3rd quartile)	Gene expression Median (1st; 3rd quartile)	Gene Expression Median (1st; 3rd quartile)
NaCl- 1ml i.p. + Rape oil -1 ml p.o.	0.57 (0.51; 0.71)	2.02E-03 (4.67E-04; 6.72E-03)	1.01 (0.84; 1.38)	6.22E-02 (8.35E-03; 2.17E-01)	5.43E-03 (1.16E-03; 1.59E-02)
VK +Rape oil	0.23 (0.17; 0.29)	3.68E-03 (1.69E-03; 7.16E-03)	1.00 (0.85; 1.17)	1.63E-02 (7.21E-03; 3.03E-02)	2.18E-02 (5.67E-03; 4.37E-02)
VK + Gabapentin	0.56 (0.4; 0.6)	1.46E-03 (9.17E-04; 3.81E-03)	0.75 (0.62; 0.9)	7.15E-03 (5.27E-03; 9.04E-03)	1.65E-02 (6.37E-03; 2.46E-02)
VK + Extract D 5 mg/kg	0.42 (0.38; 0.48)	2.85E-03 (2.44E-03; 5.59E-03)	0.56 (0.5; 0.87)	3.57E-04 (1.61E-04; 8.92E-04)	4.34E-04 (3.87E-04; 4.77E-03)
VK + Extract D 7.5 mg/kg	0.5 (0.39; 0.7)	1.64E-03 (1.50E-03; 2.86E-03)	1 (0.63; 1.1)	1.58E-02 (4.07E-04; 3.02E-01)	3.68E-03 (1.33E-03; 6.38E-03)
VK + Extract D 10 mg/kg	0.70 (0.37; 1.26)	1.70E-02 (9.11E-03; 3.28E-02)	0.59 (0.31; 0.83)	2.17E-02 (1.53E-02; 3.34E-02)	1.10E-02 (4.93E-03; 1.61E-02)
VK + Extract D 20 mg /kg	0.42 (0.25; 0.68)	4.25E-03 (4.25E-04; 1.38E-02)	0.54 (0.45; 0.65)	1.54E-03 (5.77E-04; 4.94E-03)	4.64E-03 (2.20E-03; 5.51E-03)
VK + Extract D 40 mg	0.67 (0.46; 0.89)	3.28E-03 (1.23E-03; 7.51E-03)	0.78 (0.54; 0.93)	1.88E-02 (9.55E-03; 2.04E-02)	4.34E-03 (1.37E-03; 7.40E-03)
VK + Extract B 5 mg/kg	0.44 (0.26; 0.75)	2.02E-03 (6.39E-04; 9.04E-03)	0.23 (0.19; 0.34)	1.53E-03 (7.32E-04; 3.84E-03)	1.31E-03 (7.49E-04; 5.28E-03)
VK + Extract B 7.5 mg/kg	2.29 (1.62; 3.61)	1.59E-02 (9.93E-03; 1.88E-02)	0.47 (0.33; 0.60)	8.00E-06 (0.00E+00; 4.90E-04)	6.88E-03 (2.68E-04; 2.52E-02)
VK + Extract B 10 mg/kg	0.50 (0.40; 1.00)	1.09E-02 (8.29E-03; 1.19E-02)	0.20 (0.10; 1.00)	1.34E-02 (1.28E-02; 1.47E-02)	1.29E-02 (9.70E-03; 4.08E-02)
VK + Extract B 20 mg/kg	0.54 (0.48; 0.70)	9.36E-03 (6.64E-03; 1.39E-02)	0.16 (0.07; 0.20)	1.23E-02 (9.97E-03; 1.26E-02)	2.74E-03 (7.79E-04; 5.33E-03)
VK + Extract B 40 mg/kg	0.42 (0.31; 1.42)	7.71E-03 (2.48E-03; 1.10E-02)	0.37 (0.30; 0.44)	1.45E-02 (1.02E-02; 1.93E-02)	5.99E-03 (4.35E-03; 7.89E-03)
P (Kruskal-Wallis)	p<0.001[1]	p<0.001[2]	p<0.001[3]	p<0.001[4]	p=0.0139

[1] VK+ Extract D 40 mg/kg *vs* VK+rape oil (p=0.031); VK+ Extract B 7.5 mg/kg *vs* VK+rape oil (p<0.001); VK+Extract B 7.5 mg/kg *vs* + VK+Extract D 5 mg/kg (p<0.001); VK+Extract B 7.5 mg/kg *vs* VK+Extract D 20 mg/kg (p=0.014); VK+Extract B 7.5 mg/kg *vs* VK+Extract B 5 mg/kg (p=0.014); VK+Extract B 7.5 mg/kg *vs* VK+Extract B 10 mg/kg (p=0.038)
[2] VK+ Extract D 10 mg/kg vs VK+gabapentin (p=0.024); VK+ Extract D 10 mg/kg vs VK+Extract D 7.5 mg/kg (p=0.017); VK+Extract B 7.5 mg/kg vs VK+gabapentin (p=0.010); VK+Extract B 7.5 mg/kg vs VK+Extract D 7.5 mg/kg (p=0.007)
[3] VK+Extract B 5 mg/kg vs NaCl- 1ml+ Rape oil -1 ml (p<0.001); VK+Extract B 5 mg/kg vs VK+rape oil (p<0.001); VK+Extract B 5 mg/kg vs VK+gabapentin (p=0.014); VK+Extract B 5 mg/kg vs VK+Extract D 7.5 mg/kg (p=0.014); VK+Extract B 10 mg/kg vs NaCl- 1ml+ Rape oil -1 ml (p=0.007); VK+Extract B 10 mg/kg vs VK+rape oil (p=0.008); VK+Extract B 20 mg/kg vs NaCl- 1ml+ Rape oil -1 ml (p<0.001); VK+Extract B 20 mg/kg vs VK+rape oil (p<0.001); VK+Extract B 20 mg/kg vs VK+gabapentin (p=0.005); VK+Extract B 20 mg/kg vs Extract D 7.5 mg/kg (p=0.005); VK+Extract B 40 mg/kg vs NaCl- 1ml+ Rape oil -1 ml (p=0.037); VK+Extract B 40 mg/kg vs VK+rape oil (p=0.049)
[4] VK+Extract B 7.5 mg/kg vs NaCl- 1ml+ Rape oil -1 ml (p=0.036); VK+Extract B 7.5 mg/kg vs VK+Extract D 10 mg/kg (p=0.016)

RT-PCR-Real Time PCR ELISA- enzyme-linked immunosorbent assay Vin- Vincristine - 0.1 mg/kg of body weight, intraperitoneal administration Extract D - *Cannabis sativa L extract, variety Dora*; the dose expressed as synthetic Cannabidiol, oral administration Extract B - *Cannabis sativa L extract, variety Tygra;* the dose expressed as synthetic Cannabidiol, oral administration i.p.– intraperitoneal; p.o.– per os; bw – body weight.

Table S3. TNFα expression in the brain cortex, hippocampus and lymphocytes in response to treatment with vincristine, gabapentin and CSL extracts

TNFα					
**Treatment Group**	**Hippocampus**		**Cortex**		**Lymphocytes**
	Protein [ng/ml] Median (1st; 3rd quartile)	Gene expression Median (1st; 3rd quartile)	Protein [ng/ml] Median (1st; 3rd quartile)	Gene expression Median (1st; 3rd quartile)	Gene Expression Median (1st; 3rd quartile)
NaCl- 1ml i.p. + Rape oil -1 ml p.o.	0.05 (0.02; 0.08	5.00E-06 (2.00E-06; 1.60E-05)	0.28 (0.247; 0.34)	1.43E-04 (2.80E-05; 8.13E-04)	4.92E-03 (3.17E-03; 9.69E-03)
VK +Rape oil	0.03 (0.01.; 0.13)	1.90E-05 (1.10E-05; 5.50E-05)	0.24 (0.14; 0.25)	6.40E-05 (5.90E-05; 9.20E-05)	2.67E-02 (7.37E-03; 4.25E-02)
VK + Gabapentin	0.11 (0.08; 0.2)	8.00E-06 (6.00E-06; 1.80E-05)	0.2 (0.16; 0.2)	6.30E-05 (4.70E-05; 6.70E-05)	1.31E-02 (9.46E-03; 3.34E-02)
VK + Extract D 5 mg/kg	0.08 (0.04; 0.1)	2.20E-05 (8.00E-06; 2.90E-05)	0.2 (0.18; 0.23)	4.00E-06 (1.00E-06; 2.60E-05)	2.74E-02 (1.54E-02; 4.32E-02)
VK + Extract D 7.5 mg/kg	0.1 (0.04; 0.1)	1.60E-05 (5.00E-06; 2.70E-05)	0.3 (0.23; 0.3)	3.23E-04 (5.00E-06; 7.11E-04)	2.40E-02 (1.12E-02; 4.12E-02)
VK + Extract D 10 mg/kg	0.08 (0.05; 0.09)	6.50E-05 (5.70E-05; 1.09E-04)	0.36 (0.30; 0.42)	1.13E-04 (9.40E-05; 1.66E-04)	2.56E-02 (1.54E-02; 9.05E-02)
VK + Extract D 20 mg /kg	0.03 (0.02; 0.07)	5.30E-05 (9.00E-06; 6.70E-05)	0.16 (0.06; 0.17)	7.00E-06 (4.00E-06; 3.60E-05)	2.60E-02 (1.75E-02; 6.71E-02)
VK + Extract D 40 mg	0.13 (0.09; 0.17)	1.80E-05 (7.00E-06; 6.10E-05)	0.16 (0.09; 0.21)	8.80E-05 (3.80E-05; 1.31E-04)	1.46E-02 (6.61E-03; 3.00E-02)
VK + Extract B 5 mg/kg	0.05 (0.04; 0.05)	2.00E-05 (7.00E-06; 8.10E-05)	0.05 (0.03; 0.06)	1.60E-05 (4.00E-06; 2.80E-05)	1.51E-02 (5.78E-03; 2.45E-02)
VK + Extract B 7.5 mg/kg	0.33 (0.21; 0.43)	1.17E-04 (9.40E-05; 1.88E-04)	0.09 (0.05; 0.17)	2.00E-06 (0.00E+00; 3.00E-06)	1.08E-02 (7.51E-03; 1.89E-02)
VK + Extract B 10 mg/kg	0.20 (0.20; 0.19)	1.00E-04 (7.80E-05; 1.35E-04)	0.09 (0.03; 0.22)	2.16E-04 (2.05E-04; 3.08E-04)	2.51E-02 (1.95E-02; 4.14E-02)
VK + Extract B 20 mg/kg	0.19 (0.16; 0.20)	6.80E-05 (3.70E-05; 1.00E-04)	0.03 (0.01; 0.10)	7.20E-05 (4.10E-05; 1.20E-04)	7.87E-03 (5.80E-03; 9.48E-03)
VK + Extract B 40 mg/kg	0.18 (0.08; 0.24)	3.10E-05 (9.00E-06; 1.02E-04)	0.02 (0.01; 0.04)	9.20E-05 (5.30E-05; 1.03E-04)	1.95E-02 (8.85E-03; 2.37E-02)
P (Kruskal-Wallis)	p<0.001[1]	p<0.001[2]	p<0.001[3]	p<0.001[4]	p=0.033

[1] VK+ Extract B 7.5 mg/kg vs NaCl- 1ml+ Rape oil -1 ml (p=0.004); VK+ Extract B 7.5 mg/kg vs VK+Rape oil (p=0.006); VK+ Extract B 7.5 mg/kg vs VK+ Extract D 5 mg/kg (p=0.006); VK+ Extract B 7.5 mg/kg vs VK+ Extract D 7.5 mg/kg (p=0.025); VK+ Extract B 7.5 mg/kg vs VK+ Extract D 10 mg/kg (p=0.017); VK+ Extract B 7.5 mg/kg vs VK+ Extract D 20 mg/kg (p=0.001); VK+ Extract B 7.5 mg/kg vs VK+ Extract B 5 mg/kg (p=0.004)
[2]VK+ Extract B 7.5 mg/kg vs NaCl- 1ml+ Rape oil -1 ml (p=0,002); VK+ Extract B 7.5 mg/kg vs VK+gabapentin (p=0,001); VK+ Extract B 7.5 mg/kg vs VK+ Extract D 7.5 mg/kg (p=0,014); VK+ Extract B 10 mg/kg vs NaCl- 1ml+ Rape oil -1 ml (p=0,026); VK+ Extract B 10 mg/kg vs VK+gabapentin (p=0,008)
[3] VK+ Extract B 5 mg/kg vs NaCl- 1ml+ Rape oil -1 ml (p=0,005); VK+ Extract B 5 mg/kg vs VK+ Extract D 7.5 mg/kg (p=0,008); VK+ Extract B 5 mg/kg vs VK+ Extract D 10 mg/kg (p<0.001); VK+ Extract B 10 mg/kg vs NaCl- 1ml+ Rape oil -1 ml (p=0,001); VK+ Extract B 10 mg/kg vs VK+Rape oil (p=0,044); VK+ Extract B 10 mg/kg vs VK+gabapentin (p=0,048); VK+ Extract B 10 mg/kg vs Extract D 7.5 mg/kg (p=0,002); VK+ Extract B 10 mg/kg vs Extract D 10 mg/kg (p<0.001); VK+ Extract B 20 mg/kg vs NaCl- 1ml+ Rape oil -1 ml (p<0.001); VK+ Extract B 20 mg/kg vs vs VK+Rape oil (p=0,012); VK+ Extract B 20 mg/kg vs VK+gabapentin (p=0,012); VK+ Extract B 20 mg/kg vs Extract D 7.5 mg/kg (p<0.001); VK+ Extract B 20 mg/kg vs Extract D 10 mg/kg (p<0.001)
[4]Extract B 10 mg/kg vs Extract D 5 mg/kg (p=0,002); Extract B 10 mg/kg vs Extract B 5 mg/kg (p=0,014); Extract B 10 mg/kg vs Extract B 7.5 mg/kg (p=0,006)

RT-PCR-Real Time PCR ELISA- enzyme-linked immunosorbent assay Vin- Vincristine - 0.1 mg/kg of body weight, intraperitoneal administration Extract D - *Cannabis sativa L* extract, variety Dora; the dose expressed as synthetic Cannabidiol, oral administration Extract B - *Cannabis sativa L* extract, variety Tygra; the dose expressed as synthetic Cannabidiol, oral administration i.p.– intraperitoneal; p.o.– per os; bw – body weight.
